# Frequency‐dependent signal and noise in spectroscopic x‐ray imaging

**DOI:** 10.1002/mp.14160

**Published:** 2020-04-22

**Authors:** Jesse Tanguay, Jinwoo Kim, Ho Kyung Kim, Kris Iniewski, Ian A. Cunningham

**Affiliations:** ^1^ Department of Physics Ryerson University Toronto Ontario M5B 2K3 Canada; ^2^ School of Mechanical Engineering Pusan National University Busan 609‐735 Republic of Korea; ^3^ Redlen Technologies Saanichton British Columbia Canada; ^4^ Imaging Research Laboratories, Robarts Research Institute Western University London Ontario Canada; ^5^ Department of Medical Biophysics, Schulich School of Medicine & Dentistry Western University London Ontario Canada; ^6^ Biomedical Engineering Western University London Ontario Canada

**Keywords:** detective quantum efficiency, dual-energy imaging, noise power spectrum, photon-counting, spectroscopic x-ray imaging

## Abstract

**Purpose:**

We present a new framework for theoretical analysis of the noise power spectrum (NPS) of photon‐counting x‐ray detectors, including simple photon‐counting detectors (SPCDs) and spectroscopic x‐ray detectors (SXDs), the latter of which use multiple energy thresholds to discriminate photon energies.

**Methods:**

We show that the NPS of SPCDs and SXDs, including spatio‐energetic noise correlations, is determined by the joint probability density function (PDF) of deposited photon energies, which describes the probability of recording two photons of two different energies in two different elements following a single‐photon interaction. We present an analytic expression for this joint PDF and calculate the presampling and digital NPS of CdTe SPCDs and SXDs. We calibrate our charge sharing model using the energy response of a cadmium zinc telluride (CZT) spectroscopic x‐ray detector and compare theoretical results with Monte Carlo simulations.

**Results:**

Our analysis shows that charge sharing increases pixel signal‐to‐noise ratio (SNR), but degrades the zero‐frequency signal‐to‐noise performance of SPCDs and SXDs. In all cases considered, this degradation was greater than 10%. Comparing the presampling NPS with the sampled NPS showed that degradation in zero‐frequency performance is due to zero‐frequency noise aliasing induced by charge sharing.

**Conclusions:**

Noise performance, including spatial and energy correlations between elements and energy bins, are described by the joint PDF of deposited energies which provides a method of determining the photon‐counting NPS, including noise‐aliasing effects and spatio‐energetic effects in spectral imaging. Our approach enables separating noise due to x‐ray interactions from that associated with sampling, consistent with cascaded systems analysis of energy‐integrating systems. Our methods can be incorporated into task‐based assessment of image quality for the design and optimization of spectroscopic x‐ray detectors.

## 1 Introduction

Recent technological innovations have led to photon‐counting x‐ray imaging detectors that are sensitive to individual x‐ray photon interactions and can be used to estimate the spectral distribution of interacting x‐ray quanta in spectral computed tomography (CT) and other imaging applications. One component of this research effort is the development of analytic models of detector performance that provide physical insight and understanding of relationships between basic physics, detector design and performance, and image quality. They are complementary to numerical Monte Carlo studies and may identify upper limits of performance and benchmarks for comparison.

Models are an important part of detector design to maximize visual detection of image structures. For example, the detectability index $d^{\prime }$ describes the signal‐to‐noise ratio (SNR) with which a structure can be visualized in a noise‐limited image and it is generally accepted that optimal detector design will maximize this index.^1^ For the detection of an image structure having Fourier transform *S*(*u*) relative to a uniform background, $d^{\prime }$ is given by^1^

(1) $$d^{\prime 2}=\int \frac{{\left|S\left(u\right)\right|}^{2}{\text{MTF}}^{2}\left(u\right)}{W(u)}du$$

where *u* represents spatial frequency, MTF is the detector modulation transfer function, and *W* is the image Wiener noise power spectrum (NPS). An understanding of design characteristics that determine the frequency‐dependent shape of the MTF and NPS enable optimization of $d^{\prime }$ .

Spatial‐domain calculations of the detectability index are possible using covariance matrices. They can be more compehensive than Fourier methods, but are generally far more complex and provide the same results as Fourier‐based methods for shift‐invariant systems with wide‐sense stationary (WSS) noise processes.^2^, ^3^ Fourier methods are widely used by the image‐science community when a linear and shift‐invariant (LSI) approximation is acceptable.^4^ These include the MTF to describe spatial resolution, Wiener NPS to describe image noise, noise equivalent quanta (NEQ) to describe SNR, and detective quantum efficiency (DQE).^1^, ^5^, ^6^, ^7^ The importance of Fourier methods is emphasized by the IEC who established measurement standards^1^ and its use is mandated by the US Food and Drug Administration for new‐product 510(k) application submissions.^8^

Fourier‐based linear‐systems theory has been adapted to describe quantum‐based imaging detectors. Sometimes called cascaded systems analysis (CSA), such approaches are widely used in analytic models of the Fourier‐based metrics described above.^9^, ^10^, ^11^, ^12^, ^13^ For example, task‐based performance with contrast‐enhanced breast imaging,^14^, ^15^, ^16^ digital tomosynthesis, and cone‐beam computed tomography (CT)^17^, ^18^, ^19^, ^20^ are described using these approaches. Cascaded systems analysis models have identified physical reasons for several key performance limitations,^21^, ^22^ including frequency‐dependent secondary quantum sinks,^11^ noise aliasing, reabsorption of scatter and characteristic emission photons,^13^ and the stochastic nature of scintillator and phosphor lag.^23^, ^24^ Siewerdsen *et al*.^25^ identified conditions required for early cesium‐iodide flat‐panel detectors to provide DQE performance superior to film‐screen systems. Zhao *et al*.^26^ showed that amorphous selenium with avalanche gain can improve the DQE across all spatial frequencies in fluoroscopic applications. One useful aspect of CSA is the separation of x‐ray interaction physics from sampling and aliasing issues in digital detectors. Separation of the presampling NPS from digital NPS shows that noise aliasing can reduce the DQE of many detectors by up to 40% at the sampling cutoff frequency^27^ and explains why selenium‐based detectors generally have a high‐frequency noise structure.^22^

Studies of the frequency‐dependent performance of photon‐counting detectors are complicated due to energy‐based thresholding. Acciavatti et al.^28^, ^29^ described one of the first DQE models of single‐photon‐counting detectors (SPCDs) that use an energy threshold. They interpreted the point‐spread function (PSF) of conventional energy‐integrating detectors (EIDs) as the probability per unit area of counting a photon in otherwise equivalent SPCDs. This interpretation predicts equal MTFs for SPCDs and EIDs, which has been challenged by more recent work^30^, ^31^ and has not been validated by simulation or experiment. It neglects the statistical nature of charge sharing (or optical scatter) between elements, something that is responsible for much of the frequency dependence of the DQE with EID systems.^12^ In addition, reabsorption of characteristic emissions and scatter photons was specifically ignored in this work. Reabsorption plays a dominant role in the DQE frequency shape of high‐Z detectors including CsI‐ and CdTe‐based systems, requiring a parallel‐cascades approach (or equivalent) to describe cross correlations in the NPS.^13^

Stierstorfer et al.^32^, ^33^ introduced a Monte Carlo (MC) approach for computing the frequency‐dependent DQE of SPCDs. They expressed the DQE in terms of probabilities of counting photons in elements neighboring those in which primary interactions occur. This approach does not provide theoretical access to the presampling NPS, and therefore does not provide the same level of insight into image noise and noise aliasing as CSA of EIDs.

Persson et al.^34^ applied the concept of the DQE to spectroscopic x‐ray detectors (SXDs), accounting for spatio‐energetic noise correlations^35^, ^36^ caused by charge sharing, which occurs when the energy from a single x‐ray is distributed over multiple detector elements. An MC approach was used to simulate the DQE for element sizes and energies relevant for CT imaging. They also did not address the presampling NPS or noise‐aliasing issues.

Michel et al.^37^, ^38^ modeled the zero‐frequency DQE of SPCDs and introduced the idea of multiplicity, which is the number of counts recorded per interacting photon. They showed it is possible to have a multiplicity greater than unity in the presence of charge sharing, resulting in a degraded DQE. The zero‐frequency DQE was expressed mathematically in terms of statistical moments of the multiplicity. Koenig et al.^39^ and Ji et al.^40^ used this approach to investigate the zero‐frequency DQE of cadmium telluride (CdTe) detectors, but the relationship between the DQE and the multiplicity was not validated against simulations or experiment. Other modeling works include those of Taguchi et al.,^35^, ^36^ who modeled the effects of charge sharing on spatio‐energetic noise correlations in SXDs. A similar approach was developed by Faby et al.,^41^ but neither groups performed frequency‐dependent analyses.

Previous contributions from our group^30^, ^42^, ^43^, ^44^ described the MTF and NPS of SPCDs and SXDs, including presampling metrics. In particular, it was discovered thresholding could be described using the CSA approach by propagating the probability density function (PDF) of prethresholding, presampling detector signals. In addition, the NPS is related to the joint PDF of prethresholding, presampling signals generated in two detector elements as a function of the distance between the elements. This approach was used by Xu et al.,^30^ but they did not account for x‐ray fluorescence and validated their theoretical NPS using down‐sampled image data for which high‐frequency noise is dominated by noise aliasing and not noise correlations introduced by charge sharing.

Our previous contribution did not consider reabsorption of characteristic emissions or the effect of charge migration (or optical scatter), both of which are known to have important consequences on the frequency‐dependent DQE of EID systems. In this work, we build on previous results and present an analytic framework for modeling frequency‐dependent noise in SPCDs and SXDs. Our framework enables analysis of both presampling and sampled NPS, thereby providing consistency with models of EIDs. We combine our formalism with a probabilistic model of photoelectric (PE) interactions in CdTe. We demonstrate that the combination of charge sharing and sampling leads to zero‐frequency noise aliasing, reducing the zero‐frequency performance of SPCDs and SXDs.

## 2 Theory

We first summarize a high‐level model of signal transfer through photon‐counting detectors and then present an expression for the presampling NPS, including spatio‐energetic noise correlations. We present a model of photoelectric (PE) interactions in a photon‐counting x‐ray imaging detector as a parallel cascade of image‐forming processes. We use this model to derive an analytic expression for the frequency‐dependent NPS including both presampling and sampled NPS. To our knowledge, this is the first physics‐based analytic model of frequency‐dependent noise in photon‐counting x‐ray imaging detectors accounting for charge sharing and fluorescence. We assume linearity between input and output count rates and WSS noise processes. Linearity requires low count rates such that pulse pile‐up can be ignored, which may be a good approximation for state‐of‐the‐art photon‐counting detectors in applications for breast imaging, radiography, fluoroscopy, and angiography, but not likely for computed tomography (CT). A list of parameters used in this work is given in Table 6 in Appendix A.

### 2.1 Photon‐counting signal

**Figure 1:**
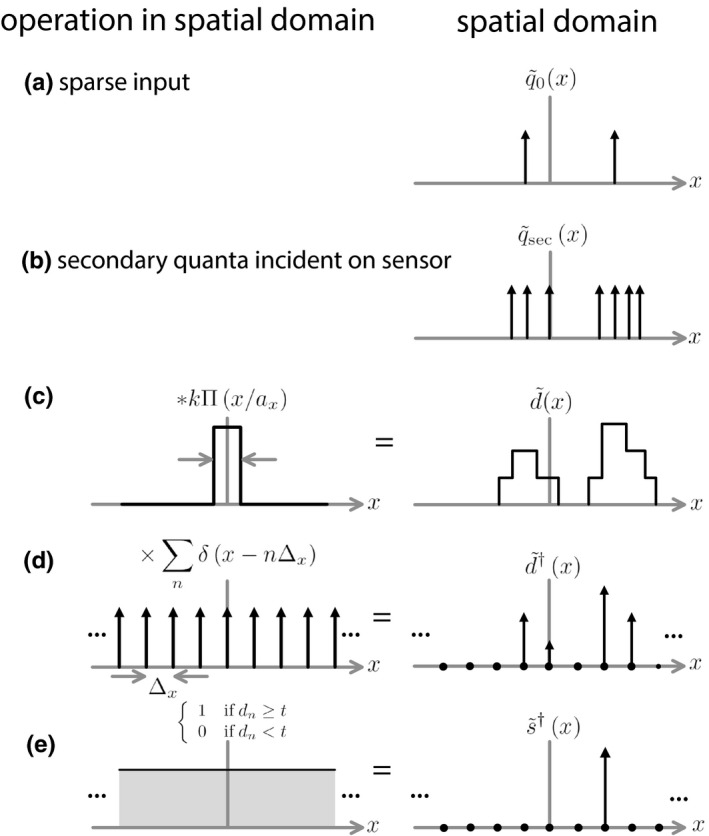
One‐dimensional schematic representation of the process of converting a sparse distribution of incident x‐ray quanta ( ${\tilde{q}}_{0}$ ) to secondary quanta such as liberated charges in a photoconductor ( ${\tilde{q}}_{\sec}$ ), to the detector presampling signal $\tilde{d}$ , and then to the thresholded signal ${\tilde{d}}^{†}$ . The superscript † indicates a function consisting of a uniform sequence of delta functions scaled by discrete detector values.

What follows here is an extension of the work presented by Tanguay et al.^43^ who described signal transfer through photon‐counting detectors using the model illustrated in Fig. 1. The model starts with a sparse distribution of x‐ray quanta incident on the x‐ray converter (a) where each photon is represented by a Dirac *δ* function. Photons that interact with the x‐ray converter produce electron‐hole (e‐h) pairs that drift across the converter under an electric field, inducing charge on collecting electrodes.^45^, ^46^ The second row (b) consists of *δ* functions indicating points at which charge carriers induce a signal on collecting electrodes. Induced charge is integrated over the element aperture (c), producing a voltage pulse from which photon energy is estimated. In Fig. 1, $\tilde{d}\left(x\right)$ represents a presampling, prethresholding signal that is proportional to the total number of charges integrated in an element centered at *x* for all possible *x*; ${\tilde{d}}^{†}\left(x\right)$ is the sampled signal represented as scaled *δ* functions at actual detector‐element centers; and ${\tilde{s}}^{†}\left(x\right)$ is the signal after thresholding.

**Figure 2:**
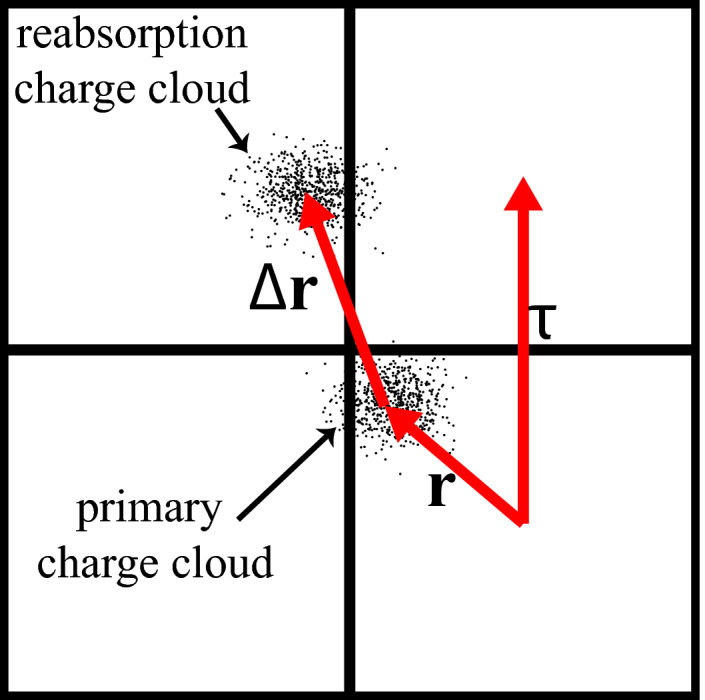
Schematic illustration of an x‐ray interaction at position **r** relative to a specified element and reabsorption of fluorescence. [Color figure can be viewed at wileyonlinelibrary.com]

An image is produced by summing ${\tilde{s}}^{†}\left(x\right)$ for all interactions. The average number of counts recorded in energy bin *j* having lower and upper energies $l_{j}$ and $u_{j}$ of a specified element is Ref. [31, 44]

(2) $${\bar{c}}_{j}={\bar{q}}_{0}\int_{l_{j}}^{u_{j}}\int_{A}p_{\varepsilon }\left(\varepsilon ;r\right)d^{2}rd\varepsilon$$

where *ɛ* [keV] represents deposited photon energy and is assumed proportional to $\tilde{d}$ , and $p_{\varepsilon }\left(\varepsilon ;r\right)$ [keV ${}^{-1}$ ] represents the PDF of *ɛ* for a primary x‐ray incidence at vector **r** relative to the element center (Fig. 2). Integration of $p_{\varepsilon }\left(\varepsilon ;r\right)$ over detector total area *A* and bin energy describes the probability of the interacting photon contributing a count to the detector element (neglecting pulse pile‐up). Charge sharing (see Fig. 2), spectral distortions, electronic noise, scatter reabsorption etc. all contribute to the PDF. In Eq. (2), the integral with respect to **r** sums photons detected at all possible element locations. This approach accounts for spreading of photon energy over multiple elements, for example, three elements, because different elements simply correspond to different values of **r**.

This is the first important observation. It shows that the PDF $p_{\varepsilon }\left(\varepsilon ;r\right)$ (when integrated over the energy bin) represents the detector point‐spread function, and its Fourier transform therefore gives the presampling MTF.^31^, ^44^

### 2.2 Photon‐counting noise

Statistical correlations between detector elements result from spreading of photon energy across multiple elements by charge sharing, fluorescence reabsorption and possibly other processes. In systems with multiple energy bins, these may lead to noise correlations between images produced from different energy bins, that is, spatio‐energetic noise correlations.^35^, ^36^

Using an approach similar to above, we show in Appendix B that the presampling cross covariance between the number of counts in energy bins *i* and *j* of two detector elements separated by vector ***τ*** is given by

(3) $$K_{i,j}\left(\tau \right)={\bar{q}}_{0}A\int_{l_{i}}^{u_{i}}\int_{l_{j}}^{u_{j}}p_{\varepsilon ,\varepsilon^{\prime }}\left(\varepsilon ,\varepsilon^{\prime };\tau \right)d\varepsilon d\varepsilon^{\prime }$$

where ${\bar{q}}_{0}A\;=\;{\bar{N}}_{0}$ represents the total number of quanta incident on the detector during image acquisition and $p_{\varepsilon ,\varepsilon^{\prime }}\left(\varepsilon ,\varepsilon^{\prime };\tau \right)$ represents the joint PDF of *ɛ* and $\varepsilon^{\prime }$ which describes the probability density of recording energy *ɛ* in one element while simultaneously recording energy $\varepsilon^{\prime }$ in a second element displaced from the first by ***τ*** given one incident photon. We refer to $p_{\varepsilon ,\varepsilon^{\prime }}\left(\varepsilon ,\varepsilon^{\prime };\tau \right)$ as the joint PDF of deposited energies which is a presampling metric and accounts for all possible locations of x‐ray incidence relative to detector elements, including situations where: no energy is deposited in either element; energy is deposited in only one element; or energy is deposited in both. In addition, while not immediately obvious, $p_{\varepsilon ,\varepsilon^{\prime }}\left(\varepsilon ,\varepsilon^{\prime };\tau \right)$ accounts for situations where more than two photons are counted, that is, triple counting, which may occur for the situation illustrated in Fig. 2. For the scenario illustrated in Fig. 2, the covariance describes the joint variability of any pair of detector elements, but not the joint variability of three or more elements, which would require the use of higher moment statistics. We are not aware of evidence that suggests the need for analysis of higher moment descriptions of noise correlations.

The presampling autocovariance of energy bin *j* is obtained by setting *i* = *j* in Eq. (3). For the case of completely overlapping elements, ***τ*** = 0 and $p_{\varepsilon ,\varepsilon^{\prime }}\left(\varepsilon ,\varepsilon^{\prime };\tau \right)$ reduces to

(4) $$p_{\varepsilon ,\varepsilon^{\prime }}\left(\varepsilon ,\varepsilon^{\prime };\tau \right){|}_{\tau =0}=\delta \left(\varepsilon -\varepsilon^{\prime }\right)p_{\varepsilon }\left(\varepsilon \right)$$

and the cross covariance as

(5) $$K_{i,j}{\left(\tau \right)|}_{\tau =0}=\delta_{\mathrm{ij}}{\bar{c}}_{j}$$

where $\delta_{\mathrm{ij}}$ represents the Kronecker delta, equal to unity when *i*=*j* and zero otherwise. Equation (5) is a good approximation when large numbers of photons are incident on detector elements.^47^ For the case of an SPCD with a single, open energy bin, Eq. (5) shows that the number of counts in detector elements remains Poisson distributed, despite the fact that charge sharing occurs. We test this prediction using the MC calculations described in Section 3.B.

This relationship between the joint PDF $p_{\varepsilon ,\varepsilon^{\prime }}\left(\varepsilon ,\varepsilon^{\prime };\tau \right)$ and the cross covariance in Eq. (3) is the second important contribution. It is a general result that does not depend on a specific model or methodology used to obtain the joint PDF. In Section 2.D we describe an analytic model of our detector, but it can also be obtained by Monte Carlo methods.

The digital NPS in a photon‐counting image with a single energy bin must include contributions from noise aliasing resulting from sampling in addition to contributions described by the presampling NPS and is given by Ref. [48]

(6) $$W_{\mathrm{dig},j}\left(u\right)=W_{j}\left(u\right)+\sum_{n=1}^{\infty }\sum_{m=1}^{\infty }W_{j}\left(u\pm u_{\mathrm{nm}}\right)$$

having units ${\text{mm}}^{2}$ where $u_{\mathrm{nm}}\;=\;\left(n/\Delta_{x},m/\Delta_{y}\right)$ and $\Delta_{x}$ and $\Delta_{y}$ represent element spacings in *x* and *y* directions, respectively, and $W_{j}\left(u\right)$ represents the presampling NPS given by

(7) $$W_{j}\left(u\right)={\bar{q}}_{0}A\cdot \text{FT}\left\{\int_{l_{j}}^{u_{j}}\int_{l_{j}}^{u_{j}}p_{\varepsilon ,\varepsilon^{\prime }}\left(\varepsilon ,\varepsilon^{\prime };\tau \right)d\varepsilon d\varepsilon^{\prime }\right\}$$

with units ${\text{mm}}^{2}$ where FT{·} represents the Fourier transform. Similarly, the digital cross NPS of energy bins *i* and *j*, which also includes contributions from noise aliasing, is given by

(8) $$W_{\mathrm{dig},i,j}\left(u\right)=W_{i,j}\left(u\right)+\sum_{n=1}^{\infty }\sum_{m=1}^{\infty }W_{i,j}\left(u\pm u_{\mathrm{nm}}\right)$$

where $W_{i,j}\left(u\right)$ represents the presampling cross NPS of bins *i* and *j*:

(9) $$W_{i,j}\left(u\right)={\bar{q}}_{0}A\cdot \text{FT}\left\{\int_{l_{i}}^{u_{i}}\int_{l_{j}}^{u_{j}}p_{\varepsilon ,\varepsilon^{\prime }}\left(\varepsilon ,\varepsilon^{\prime };\tau \right)d\varepsilon d\varepsilon^{\prime }\right\}.$$

The digital NPS and cross NPS are defined for frequencies less than the Nyquist cutoff frequency.^48^

Equations (6), (7), (8), (9) are the third important contribution and show that the NPS of any one energy bin and the cross NPS of any two bins are fully determined by $p_{\varepsilon ,\varepsilon^{\prime }}\left(\varepsilon ,\varepsilon^{\prime };\tau \right)$ .

### 2.3 Pixel SNR, zero‐frequency DQE, and multiplicity

#### 2.3.1 Pixel SNR

Pixel SNR is used by some investigators to describe detector performance, but this is meaningful and gives the zero‐frequency DQE value only when pixels are sufficiently large to ensure no significant charge sharing or fluorescence reabsorbtion between pixels, and this is generally not the case. The pixel SNR of an SPCD normalized by the SNR of an ideal detector that preserves the SNR of the incident quanta is given by

(10) $$\frac{{\text{SNR}}^{2}}{{\text{SNR}}_{\mathrm{ideal}}^{2}}=\frac{{\bar{c}}^{2}}{{\bar{q}}_{0}a\sigma_{c}^{2}}=\frac{\bar{c}}{{\bar{q}}_{0}a}$$

where $\sigma_{c}^{2}$ represents the variance in the number of counts in the element and ${\text{SNR}}_{\mathrm{ideal}}^{2}\;=\;{\bar{q}}_{0}a$ . Equation (10) predicts an increase in pixel SNR with increasing multiplicity, which could be greater than the quantum efficiency, resulting in a pixel SNR greater than that of an ideal photon counter that records exactly one count per interaction.

#### 2.3.2 DQE(0)

**Figure 3:**
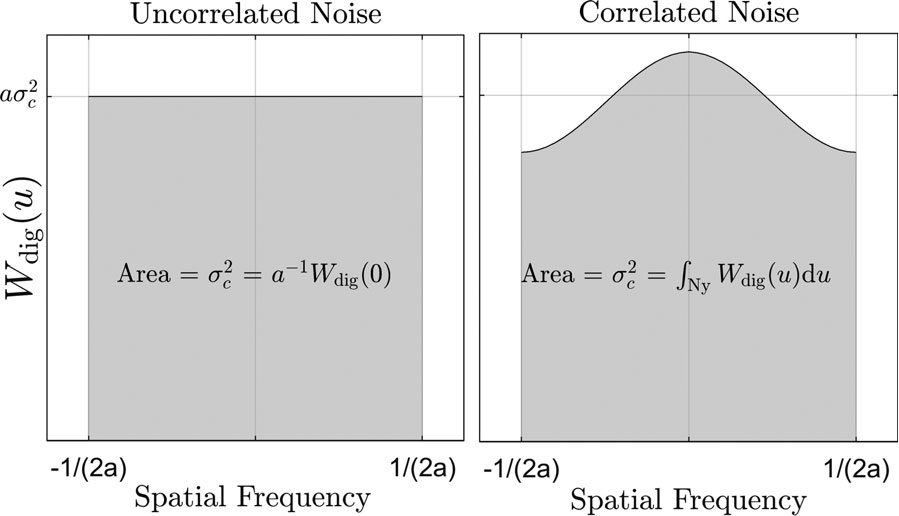
A one‐dimensional schematic illustration of the relationship between image variance ( $\sigma_{c}^{2}$ ) and $W_{\mathrm{dig}}\left(u\right)$ for uncorrelated and correlated image noise. For a fixed variance, introducing noise correlations must increase zero‐frequency noise. The parameter *a* represents the pixel area.

The zero‐frequency (i.e., large area) performance of an imaging system is described by the zero‐frequency DQE:^5^

(11) $$\text{DQE}\left(0\right)=\frac{{\bar{c}}^{2}}{{\bar{q}}_{0}W_{\mathrm{dig}}\left(0\right)}=\frac{1}{{\text{NNPS}}_{\mathrm{dig}}\left(0\right)}$$

where ${\text{NNPS}}_{\mathrm{dig}}\left(u\right)={\bar{q}}_{0}W_{\mathrm{dig}}\left(u\right)/{\bar{c}}^{2}$ represents the normalized digital NPS. Equations (10) and (11) are equivalent when $\sigma_{c}^{2}\;=\;a^{-1}W_{\mathrm{dig}}\left(0\right)$ . Since $W_{\mathrm{dig}}\left(u\right)$ has a global maximum at **u** = 0 (all physical scattering and image‐blurring mechanisms pass any uncorrelated NPS component unchanged and any correlated component scaled by the (squared) scatter transfer function which always decreases with increasing frequency^9^) and $\sigma_{c}^{2}$ is equal to the integral of $W_{\mathrm{dig}}\left(u\right)$ over frequency, the only possible scenario for which $\sigma_{c}^{2}\;=\;a^{-1}W_{\mathrm{dig}}\left(0\right)$ is that of uncorrelated noise. Figure 3 illustrates $W_{\mathrm{dig}}\left(u\right)$ for correlated and uncorrelated noise with the same pixel variance. Correlated noise reduces $W_{\mathrm{dig}}\left(u\right)$ near the Nyquist frequency, which must be accompanied by an increase in zero‐frequency noise to preserve the pixel variance. It follows that

(12) $$\text{DQE}\left(0\right)\leq \frac{{\text{SNR}}^{2}}{{\text{SNR}}_{\mathrm{ideal}}^{2}}$$

where equality is only achieved when noise is uncorrelated. Therefore, in general, an increase in pixel SNR does not necessarily correspond to an increase in DQE(0). Our results below show that zero‐frequency noise aliasing is the cause of increased zero‐frequency noise in SPCDs and SXDs. This is a counter‐intuitive result that is discussed further in the Discussion section.

#### 2.3.3 Multiplicity

We show here that the formalism presented above yields the same zero‐frequency DQE as that of the multiplicity framework introduced by Michel et al.^37^ We first rewrite Eq. (11) as^12^, ^37^

(13) $$\text{DQE}\left(0\right)=\frac{{\bar{c}}^{2}}{{\bar{q}}_{0}a\sum_{l,n=0}^{\infty }K_{\pm l,\pm n}}$$

where $K_{\pm l,\pm n}$ represents the covariance of two elements separated by $\tau_{\pm l,\pm n}\;=\;\left(\pm l\Delta ,\;\pm \;n\Delta \right)$ where Δ represents the pixel spacing. The multiplicity is equal to the average number of detected photons per interaction and is related to $\bar{c}$ :

(14) $$\bar{m}=\frac{\bar{c}}{{\bar{q}}_{0}a\alpha }=\frac{1}{a\alpha }\int_{\varepsilon_{t}}^{\infty }\int_{A}p_{\varepsilon }\left(\varepsilon ;r\right)d^{2}rd\varepsilon$$

where $\varepsilon_{t}$ represents the energy threshold used to identify individual photon interactions. We show in Appendix C that the second statistical moment of the multiplicity ( $\bar{m^{2}}$ ) can be expressed in terms of $K_{\pm l,\pm n}$ :

(15) $$\bar{m^{2}}=\frac{1}{{\bar{q}}_{0}a\alpha }\sum_{l,n=0}^{\infty }K_{\pm l,\pm n}$$

Combining Eqs. (13) and (15) yields

(16) $$\text{DQE}\left(0\right)=\alpha \frac{{\bar{m}}^{2}}{\bar{m^{2}}}$$

where $\bar{m^{2}}$ can be expressed in terms of the joint distribution of deposited energies (see Appendix C):

(17) $$\bar{m^{2}}=\frac{1}{\alpha }\sum_{l,n=0}^{\infty }\int_{\varepsilon_{t}}^{\infty }\int_{\varepsilon_{t}}^{\infty }p_{\varepsilon ,\varepsilon^{\prime }}\left(\varepsilon ,\varepsilon^{\prime };\tau_{\pm l,\pm n}\right)d\varepsilon d\varepsilon^{\prime }.$$

Equation (16) was first presented by Michel et al.^37^ and has an analytic form similar to the Swank noise factor for energy‐integrating systems.^49^

These analytic results show that the framework presented in Sections 2.A and 2.B reduces to the multiplicity framework for zero‐frequency analysis of SPCDs, and is thus a generalization of the multiplicity approach to nonzero frequencies and to systems that use multiple energy bins.

### 2.4 Cascaded model of x‐ray interactions

**Figure 4:**
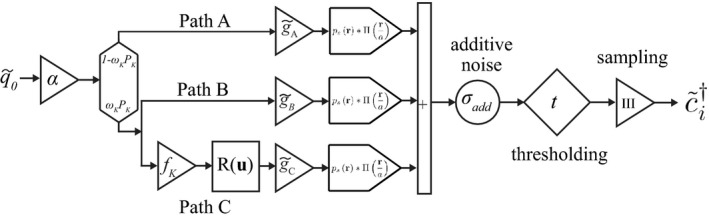
Schematic illustration of the parallel cascaded model used to describe PE interactions in single‐Z x‐ray converters.

In the preceding sections we introduced general expressions relating the autocovariance and NPS to the joint PDF of deposited energies. In this section, we develop an analytic model of this joint PDF for high‐Z photon‐counting detectors such as CdTe and CZT. The primary interaction mechanism in high‐Z materials is the PE effect. We therefore neglect reabsorption of Compton x‐rays, as described in more detail in Section 3.A. We assume a single‐Z converter using the parallel CSA approach shown in Fig. 4.^13^, ^22^, ^50^ Extension to compound semiconductors is described in Section 3.A. This model accounts for diffusion, Coulomb repulsion, reabsorption of characteristic x‐rays following interactions with K‐shell electrons, initial sizes of charge clouds, electronic noise, conversion gain, and energy thresholding. We recently used this model to calculate the large‐area gain and MTF of SPCDs and SXDs.^31^ Here, we calculate the photon‐counting NPS, which requires a model for $p_{\varepsilon ,\varepsilon^{\prime }}\left(\varepsilon ,\varepsilon^{\prime };\tau \right)$ . We first briefly summarize analytic expressions of the energy response function and large‐area gain presented by Tanguay et al.^31^ A list of parameters is given in Table 6.

#### 2.4.1 Energy response function

The energy response function for the model in Fig. 4 is given by Ref. [51, 52]

(18) $$\begin{array}{cc} p_{\varepsilon }\left(\varepsilon \right) & =\alpha \left(1-P_{K}\omega_{K}\right)p_{\varepsilon ,A}\left(\varepsilon \right)+\alpha P_{K}\omega_{K}\left(1-f_{K}\right)p_{\varepsilon ,B}\left(\varepsilon \right)\\ & +\alpha P_{K}\omega_{K}f_{K}p_{\varepsilon ,B+C}\left(\varepsilon \right) \end{array}$$

where $p_{\varepsilon ,A}\left(\varepsilon \right)$ represents the distribution of deposited energies for PE interactions for which no K‐shell photon is produced, $p_{\varepsilon ,B}\left(\varepsilon \right)$ represents the case where a K‐shell photon is produced but escapes the detector, and $p_{\varepsilon ,B+C}\left(\varepsilon \right)$ represents the case where a K‐shell photon is produced and reabsorbed. Variables $\omega_{K}$ , $P_{K}$ and $f_{K}$ represent K‐shell fluorescence yield, K‐shell participation fraction, and probability of reabsorption given the production of a K‐shell characteristic photon, respectively. Although not stated explicitly, each variable in Eq. (18) is energy dependent.

The PDFs $p_{\varepsilon ,A}\left(\varepsilon \right)$ and $p_{\varepsilon ,B}\left(\varepsilon \right)$ have the form^31^, ^51^, ^52^

(19) $$p_{\varepsilon ,X}\left(\varepsilon \right)=\frac{1}{A}\int_{A}N_{X}\left(\varepsilon ,r\right)d^{2}r$$

where *X* ∈ {*A*,*B*} and $N_{X}$ represents a normal distribution with position‐dependent mean and variance respectively given by

(20) $${\bar{\varepsilon }}_{X}\left(r\right)=E_{X}P_{\mathrm{CS}}\left(r\right)$$

(21) $$\sigma_{X}^{2}\left(r\right)={\bar{\varepsilon }}_{X}\left(r\right)+\sigma_{e}^{2}$$

where $E_{X}$ [keV] represents the total energy deposited along path *X*, $\sigma_{e}$ [keV] represents the electronic noise level, and $P_{\mathrm{CS}}\left(r\right)$ represents the probability that a charge carrier is detected in an element centered at **r**:

(22) $$P_{\mathrm{CS}}\left(r\right)=\Pi \left(\frac{r}{a}\right)*p_{\mathrm{CS}}\left(r\right)$$

where $p_{\mathrm{CS}}\left(r\right)$ [mm ${}^{-2}$ ] represents the charge‐sharing kernel normalized to unity. Equations (19), (20), (21), (22) were derived assuming the number of liberated e‐h pairs is Poisson distributed. In reality, the variance $\left(\sigma_{g}^{2}\right)$ of the number of liberated e‐h pairs is less than the corresponding mean value ( $\bar{g}$ ), in which case $\sigma_{g}^{2}=F\bar{g}$ where *F* is the Fano factor.^53^ We show in Appendix D that Eqs. (19), (20), (21), (22) also apply when *F*≪1 and $\bar{g}\gg 1$ , and are therefore good approximations for CZT and CdTe, for which $\bar{g}∼10^{4}$ and *F*∼0.1.^54^, ^55^

The PDF $p_{\varepsilon ,B\;+\;C}\left(\varepsilon \right)$ is given by^31^, ^44^

(23) $$p_{\varepsilon ,B+C}\left(\varepsilon \right)=\frac{1}{A}\int_{A}N_{B+C}\left(\varepsilon ;r,r^{\prime }\right){*}_{r^{\prime }}p_{K}\left(r^{\prime }\right){|}_{r=r^{\prime }}d^{2}r$$

where $p_{K}\left(r\right)$ represents the PDF of characteristic photon reabsorption having transfer function *R*(**u**),^13^, ^50^ and $N_{B\;+\;C}\left(\varepsilon ;r,r^{\prime }\right)$ represents a normal distribution with mean and variance given by

(24) $${\bar{\varepsilon }}_{B+C}\left(r,r^{\prime }\right)={\bar{\varepsilon }}_{A}\left(r\right)+{\bar{\varepsilon }}_{C}\left(r+r^{\prime }\right)$$

(25) $$\sigma_{B+C}^{2}\left(r,r^{\prime }\right)={\bar{\varepsilon }}_{B+C}\left(r,r^{\prime }\right)+\sigma_{e}^{2}.$$

The notation in Eq. (23) indicates that $N_{B\;+\;C}\left(\varepsilon ;r,r^{\prime }\right)$ is convolved with $p_{K}\left(r^{\prime }\right)$ while holding **r** fixed, evaluated at $r^{\prime }=r$ , and then integrated with respect to **r**. Convolution with respect to $r^{\prime }$ accounts for reabsorption of characteristic photons at sites remote from primary interactions.

#### 2.4.2 Large‐area gain

The energy‐dependent large‐area gain is obtained from integration of the energy response function over the $i^{\text{th}}$ energy bin:^31^, ^44^

(26) $$\begin{array}{cc} {\bar{G}}_{i} & =\alpha \left(1-P_{K}\omega_{K}\right)\kappa_{A,i}+\alpha P_{K}\omega_{K}\left(1-f_{K}\right)\kappa_{B,i}\\ & +\alpha P_{K}\omega_{K}f_{K}\kappa_{B+C},i \end{array}$$

where $\kappa_{X}$ is given by

(27) $$\kappa_{X,i}=\frac{A}{a}\int_{l_{i}}^{u_{i}}p_{\varepsilon ,X}\left(\varepsilon \right)d\varepsilon$$

for *X* ∈ [*A*,*B*,*B* + *C*]. The gain for an x‐ray spectrum is obtained by averaging Eq. (26) over the spectrum.

#### 2.4.3 Joint PDF of deposited energies

We calculate the joint PDF of deposited energies for the case of nonoverlapping elements, that is, $\tau \notin \left[-\frac{a_{x}}{2},\frac{a_{x}}{2}\right]\;\times \;\left[-\frac{a_{y}}{2},\frac{a_{y}}{2}\right]$ . For ***τ*** = 0, the joint PDF is given by $p_{\varepsilon }\left(\varepsilon \right)$ ; the region between ***τ*** = 0 and $\tau \notin \left[-\frac{a_{x}}{2},\frac{a_{x}}{2}\right]\;\times \;\left[-\frac{a_{y}}{2},\frac{a_{y}}{2}\right]$ can be obtained by linear interpolation without introducing errors for frequencies less $1/a_{x}$ . Following previous work^51^, ^52^ we obtain

(28) $$\begin{array}{cc} p_{\varepsilon ,\varepsilon^{\prime }}\left(\varepsilon ,\varepsilon^{\prime };\tau \right) & =\alpha \left(1-P_{K}\omega_{K}\right)p_{\varepsilon ,\varepsilon^{\prime }}^{A}\left(\varepsilon ,\varepsilon^{\prime };\tau \right)\\ & +\alpha P_{K}\omega_{K}p_{\varepsilon ,\varepsilon^{\prime }}^{B+C}\left(\varepsilon ,\varepsilon^{\prime };\tau \right) \end{array}$$

where $p_{\varepsilon ,\varepsilon^{\prime }}^{A}$ and $p_{\varepsilon ,\varepsilon^{\prime }}^{B\;+\;C}$ represent the joint PDFs of deposited energies for photons that follow path A and paths B+C, respectively. The factors $P_{K}\omega_{K}$ and $\left(1-P_{K}\omega_{K}\right)$ represent probabilities that a PE interaction does or does not result in the production of a K‐shell characteristic x ray, respectively.

Path A represents PE interactions for which there is no characteristic emission. In this case, all photon energy is deposited locally but may be distributed over more than one element. To calculate $p_{\varepsilon ,\varepsilon^{\prime }}^{A}\left(\varepsilon ,\varepsilon^{\prime };\tau \right)$ , we model integration of charge in two nonoverlapping electrodes as the problem of random Poisson points in nonoverlapping intervals.^56^ To this end, we consider the probability that *n* quanta are collected in an element centered at **r** while simultaneously $n^{\prime }$ quanta are collected in an element centered at **r** + ***τ*** following an interaction at ${\tilde{r}}_{0}$ . When the elements centered at **r** and **r** + ***τ*** do not overlap, the joint PDF of *n* and $n^{\prime }$ can be modeled as a multinomial distribution with three mutually exclusive outcomes for each quantum:^47^ (a) the quantum is detected in the element centered at **r**; (b) the quantum is detected in the element centered at **r** + ***τ***; and (c) the quantum is not detected in either. In the limit of a large number of collected quanta, which must be satisfied for viable detector designs, this multinomial distribution can be approximated as the product of two Poisson distributions.^47^ Accounting for the random location of x‐ray incidence, electronic noise, and approximating the Poisson distribution as a normal distribution yields the joint PDF for path A:

(29) $$p_{\varepsilon ,\varepsilon^{\prime }}^{A}\left(\varepsilon ,\varepsilon^{\prime };\tau \right)\approx \frac{1}{A}N_{A}\left(\varepsilon ;\tau \right){*}_{\tau }N_{A}\left(\varepsilon ;\tau \right)$$

where $N_{A}\left(\varepsilon ;\tau \right)$ represents a normal distribution with mean and variance given by Eqs. (20) and (21), respectively. The convolution in Eq. (29) results from averaging over all possible locations of x‐ray incidence.

Paths B and C describe PE interactions that produce K‐shell characteristic photons, resulting in energy deposition at primary and reabsorption sites, as illustrated in Fig. 2. We let $\varepsilon_{B}$ and $\varepsilon_{C}$ represent the energy deposited from paths B and C, respectively, and when a characteristic x ray is produced, the total energy deposited is $\varepsilon \;=\;\varepsilon_{B}\;+\;\varepsilon_{C}$ . We let $p_{\varepsilon ,\varepsilon^{\prime }}^{B}\left(\varepsilon_{B},\varepsilon_{B}^{\prime };\tau |r-{\tilde{r}}_{0}\right)$ represent the joint PDF for path B given a primary interaction at ${\tilde{r}}_{0}$ , and similarly for path C. The joint PDF for paths B and C given $\varepsilon_{C}$ , $\varepsilon_{C}^{\prime }$ , and ${\tilde{r}}_{0}$ is then given by

(30) $$\begin{array}{cc} & p_{\varepsilon ,\varepsilon^{\prime }}^{B+C}\left(\varepsilon ,\varepsilon^{\prime };\tau |\varepsilon_{C},\varepsilon_{C}^{\prime },r-{\tilde{r}}_{0}\right)\\ & =p_{\varepsilon ,\varepsilon^{\prime }}^{B}\left(\varepsilon_{B},\varepsilon_{B}^{\prime };\tau |r-{\tilde{r}}_{0}\right){|}_{\varepsilon_{B}=\varepsilon -\varepsilon_{C},\varepsilon_{B}^{\prime }=\varepsilon^{\prime }-\varepsilon_{C}^{\prime }} \end{array}$$

which states that the probability of observing energy *ɛ* given $\varepsilon_{C}$ is equal to the probability of observing $\varepsilon_{B}\;=\;\varepsilon -\varepsilon_{C}$ . Averaging $p_{\varepsilon ,\varepsilon^{\prime }}^{B\;+\;C}$ over $\varepsilon_{C}$ , $\varepsilon_{C}^{\prime }$ , and ${\tilde{r}}_{0}$ yields

(31) $$\begin{array}{cc} & p_{\varepsilon ,\varepsilon^{\prime }}^{B+C}\left(\varepsilon ,\varepsilon^{\prime };\tau \right)\\ & =\frac{1}{A}\int_{A}p_{\varepsilon ,\varepsilon^{\prime }}^{B}\left(\varepsilon ,\varepsilon^{\prime };\tau |r-{\tilde{r}}_{0}\right){*}_{\varepsilon ,\varepsilon^{\prime }}p_{\varepsilon ,\varepsilon^{\prime }}^{C}\left(\varepsilon ,\varepsilon^{\prime };\tau |r-{\tilde{r}}_{0}\right)d^{2}r_{0} \end{array}$$

where ${*}_{\varepsilon ,\varepsilon^{\prime }}$ represents a 2D convolution with respect to *ɛ* and $\varepsilon^{\prime }$ and accounts for the average over $\varepsilon_{C}$ and $\varepsilon_{C}^{\prime }$ , $p_{\varepsilon ,\varepsilon^{\prime }}^{B}\left(\varepsilon ,\varepsilon^{\prime };\tau |r-{\tilde{r}}_{0}\right)\;=\;N_{B}\left(\varepsilon ;r-{\tilde{r}}_{0}\right)N_{B}\left(\varepsilon ;r-{\tilde{r}}_{0}\;+\;\tau \right)$ (as in path A), and the integral with respect to $r_{0}$ represents an average over all possible locations of primary PE interactions.

For path C, we must account for the probability of reabsorption ( $f_{K}$ ) and relocation of the characteristic photon, which leads to the following joint PDF:

(32) $$\begin{array}{cc} & p_{\varepsilon ,\varepsilon^{\prime }}^{C}\left(\varepsilon ,\varepsilon^{\prime };\tau |r-{\tilde{r}}_{0}\right)=\left(1-f_{K}\right)\delta \left(\varepsilon \right)\delta \left(\varepsilon^{\prime }\right)\\ & +f_{K}p_{K}\left(r-{\tilde{r}}_{0}\right){*}_{r}\left[N_{C}\left(\varepsilon ;r-{\tilde{r}}_{0}\right)N_{C}\left(\varepsilon ;r+\tau -{\tilde{r}}_{0}\right)\right] \end{array}$$

The first term accounts for the case where the characteristic photon is not absorbed and the second for reabsorption. Convolution with respect to **r** is a result of averaging over the location of reabsorption.

Combining Eqs. (31) and (32) yields

(33) $$\begin{array}{cc} p_{\varepsilon ,\varepsilon^{\prime }}^{B+C}\left(\varepsilon ,\varepsilon^{\prime };\tau \right) & =\frac{\left(1-f_{K}\right)}{A}N_{B}\left(\varepsilon ;\tau \right){*}_{\tau }N_{B}\left(\varepsilon^{\prime };\tau \right)\\ & +\frac{f_{K}}{A}\int_{A}\left[N_{B}\left(\varepsilon ;\tau \right)N_{B}\left(\varepsilon^{\prime };r+\tau \right)\right]\\ & {*}_{\varepsilon ,\varepsilon^{\prime }}[p_{K}\left(r\right){*}_{r}\left[N_{C}\left(\varepsilon ;\tau \right)N_{C}\left(\varepsilon^{\prime };r+\tau \right)\right]]d^{2}r \end{array}$$

which (see Appendix E) can be simplified as

(34) $$p_{\varepsilon ,\varepsilon^{\prime }}^{B+C}\left(\varepsilon ,\varepsilon^{\prime };\tau \right)=\frac{\left(1-f_{K}\right)}{A}N_{B}\left(\varepsilon ;\tau \right){*}_{\tau }N_{B}\left(\varepsilon^{\prime };\tau \right)$$

(35) $$\begin{array}{cc} & +\frac{f_{K}}{A}\int_{A}[N_{B+C}\left(\varepsilon ;\tau ,\tau -\tau^{\prime }\right)\\ & {*}_{\tau }N_{B+C}\left(\varepsilon^{\prime };\tau ,\tau -\tau^{\prime }\right)]p_{K}\left(\tau^{\prime }\right)d^{2}\tau^{\prime }. \end{array}$$

Combining Eqs. (28), (29) and (33) yields $p_{\varepsilon ,\varepsilon^{\prime }}\left(\varepsilon ,\varepsilon^{\prime };\tau \right)$ for the model illustrated in Fig 4.

#### 2.4.4 Presampling autocovariance function

Combining $p_{\varepsilon ,\varepsilon^{\prime }}\left(\varepsilon ,\varepsilon^{\prime };\tau \right)$ from the preceding section with Eq. (3) yields the presampling autocovariance between energy bins *i* and *j*:

(36) $$\begin{array}{cc} K_{i,j}\left(\tau \right) & ={\bar{q}}_{0}{\left\langle \alpha \left(1-P_{K}\omega_{K}\right)\Phi_{A,i}\left(\tau \right){*}_{\tau }\Phi_{A,j}\left(\tau \right)\right\rangle }_{{\bar{q}}_{0}}+{\bar{q}}_{0}{\left\langle \alpha P_{K}\omega_{K}\left(1-f_{K}\right)\Phi_{B,i}\left(\tau \right){*}_{\tau }\Phi_{B,j}\left(\tau \right)\right\rangle }_{{\bar{q}}_{0}}\\ & +{\bar{q}}_{0}{〈\alpha P_{K}\omega_{K}f_{K}\int_{A}\Phi_{B+C,i}\left(\tau ,\tau -\tau^{\prime }\right){*}_{\tau }\Phi_{B+C,j}\left(\tau ,\tau -\tau^{\prime }\right)p_{K}\left(\tau^{\prime }\right)d^{2}\tau^{\prime }〉}_{{\bar{q}}_{0}} \end{array}$$

where

(37) $$\Phi_{X,i}\left(\tau \right)=\int_{l_{i}}^{u_{i}}N_{X}\left(\varepsilon ;\tau \right)d\varepsilon$$

for *X* ∈ {*A*,*B*} and

(38) $$\Phi_{B+C,i}\left(\tau ,\tau^{\prime }\right)=\int_{l_{i}}^{u_{i}}N_{B+C}\left(\varepsilon ;\tau ,\tau^{\prime }\right)d\varepsilon$$

where $N_{B+C}\left(\varepsilon ;\tau ,\tau^{\prime }\right)$ represents a normal distribution with mean and variance given by Eqs (24) and (25), respectively.

The first term in Eq. (36) accounts for photons that follow path A in Fig. 4, the second and third terms account for photons that follow paths B and C, with the third term accounting for noise correlations between paths B and C. The shift by $\tau^{\prime }$ in the integrand of the third term of Eq. (36) accounts for the relocation of characteristic x rays relative to the sites of primary interactions. We note that the third term cannot be reduced to a simple sequence of convolutions, which is an unfortunate result of the nonlinear nature of energy thresholding.

Equation (36) represents a complex theoretical development that can be used to describe the spectral cross covariance of any single‐Z detector resulting from PE interactions.

## 3 Materials and methods

### 3.1 Application to CdTe

We used the mathematical methods described in the preceding section to model the presampling and digital NPS of a detector with the material and electrical properties of modern CZT and CdTe. Numerical implementation, imaging parameters, and our model of charge conversion, charge collection, and charge sharing are described below.

#### 3.1.1 Numerical calculation of the presampling NPS, digital NPS, and zero‐frequency DQE

The following calculations were performed for selected combinations of imaging parameters described in Section 3.A.4. We assumed a CdTe detector and calculated the presampling NPS as

(39) $$W_{i,j}\left(u\right)=\nu_{\mathrm{Cd}}W_{i,j}^{\mathrm{Cd}}\left(u\right)+\nu_{\mathrm{Te}}W_{i,j}^{\mathrm{Te}}\left(u\right)$$

where $W_{i,j}^{\mathrm{Cd}}\left(u\right)$ and $W_{i,j}^{\mathrm{Te}}\left(u\right)$ represent the noise power spectra for interactions that occur with Cd and Te, respectively, and $\nu_{\mathrm{Cd}}$ and $\nu_{\mathrm{Te}}$ represent the probabilities of interactions with Cd and Te given that an interaction occurs, respectively. We calculated $W_{i,j}^{\mathrm{Cd}}\left(u\right)$ using the following:

(40) $$W_{i,j}^{\mathrm{Cd}}\left(u\right)=\xi_{\mathrm{PE}}W_{i,j}^{\mathrm{Cd},\mathrm{PE}}\left(u\right)+\left(1-\xi_{\mathrm{PE}}\right)W_{i,j}^{\mathrm{Cd},\mathrm{Co}}\left(u\right)$$

where $\xi_{\mathrm{PE}}$ represents the probability that an interaction with Cd is PE, $W_{i,j}^{\mathrm{Cd},\mathrm{PE}}$ represents the NPS given a PE interaction with Cd, and $W_{i,j}^{\mathrm{Cd},\mathrm{Co}}$ represents the NPS given a Compton interaction; $W_{i,j}^{\mathrm{Te}}\left(u\right)$ was calculated similarly. Photoelectric noise power spectra were calculated from the autocovariance function described above. While the autocovariance model described above could be extended to include Compton interactions, Compton interactions represent less than 5 % of interactions in CdTe and CZT for the average energies of RQA5, RQA7, and RQA9 x‐ray spectra. We therefore only used path A of Fig. 4 to model Compton interactions, for which we assumed the energy deposited by a Compton interaction was equal to the energy transferred to the recoil electron.

Calculation of $W_{i,j}^{\mathrm{Cd},\mathrm{PE}}\left(u\right)$ and $W_{i,j}^{\mathrm{Te},\mathrm{PE}}\left(u\right)$ requires calculation of the corresponding presampling covariance functions. While it is possible to calculate $p_{\varepsilon ,\varepsilon^{\prime }}\left(\varepsilon ,\varepsilon^{\prime };\tau \right)$ and subsequently $K_{i,j}\left(\tau \right)$ , this requires evaluating $p_{\varepsilon ,\varepsilon^{\prime }}\left(\varepsilon ,\varepsilon^{\prime };\tau \right)$ over a rectangular domain of energies *ɛ* and $\varepsilon^{\prime }$ , which is computationally expensive. We calculated $K_{i,j}\left(\tau \right)$ directly from $\Phi_{A,i}\left(\tau \right)$ , $\Phi_{B,i}\left(\tau \right)$ , and $\Phi_{B\;+\;C,i}\left(\tau \right)$ , which are related to cumulative normal distributions, for which there are efficient computational algorithms. For $\Phi_{A,i}\left(\tau \right)$ and $\Phi_{B,i}\left(\tau \right)$ , we calculated the mean and variance by combining the charge sharing kernel from Section 3.A.2 with Eqs. (20), (21), (22). The resulting mean and variance were combined with MATLAB’s “normcdf” function to calculate $\Phi_{A,i}\left(\tau \right)$ and $\Phi_{B,i}\left(\tau \right)$ . For the third term in Eq. (36), we calculated $\Phi_{B\;+\;C,i}\left(\tau ,\tau -\tau^{\prime }\right)*\Phi_{B+C,j}\left(\tau ,\tau -\tau^{\prime }\right)$ for each value of ***τ*** and $\tau^{\prime }$ , multiplied by $p_{K}\left(\tau^{\prime }\right)$ and then integrated numerically with respect to $\tau^{\prime }$ . The function $p_{K}\left(\tau \right)$ and the reabsorption probability ( $f_{K}$ ) were calculated using the methods described by Tanguay et al.^52^

The approach described in the preceding paragraph yielded the presampling autocovariance in the region of nonoverlapping elements; the region of overlapping elements was obtained using bilinear interpolation. The resulting presampling autocovariance function was subsequently Fourier transformed to yield the presampling NPS from which the digital NPS was obtained. Equation (11) was then used to calculate the zero‐frequency DQE.

#### 3.1.2 Charge conversion, charge collection, charge sharing, and electronic noise

We assumed Poisson‐distributed conversion gain (i.e., *F* = 1), normally distributed electronic noise ( $\sigma_{e}\;=\;2$ keV), and incorporated the charge‐sharing model described below. In all cases, we assumed charge trapping and charge recombination were negligible, which is a good approximation for modern CdTe and CZT under low‐flux conditions.^57^, ^58^ While the Fano factor of CZT and CdTe is ∼0.1,^54^, ^55^ this will have a negligible effect. For example, if we ignore charge sharing and consider the average energy of the RQA7 spectrum and an electronic noise level of 2 keV, the FWHM of the photopeak with and without the Fano factor is ∼4.73 keV and ∼4.89 keV, respectively. Table 1 lists nominal material properties used in our calculations

Holes and electrons contribute to the charge integrated in a pixel electrode, but electrons drift further than holes when drifted toward the exit side of the detector. We therefore only considered the electron cloud, which was assumed to be spherically symmetric and was calculated from the following charge transport equation:

(41) $$\frac{\partial Q}{\partial t}=\frac{\mu_{e}}{8\pi \epsilon r^{2}}\frac{\partial Q^{2}}{\partial r}+\frac{\mu_{e}kT}{q_{-}}\left(\frac{\partial^{2}Q}{\partial r^{2}}-\frac{2}{r}\frac{\partial Q}{\partial r}\right)$$

where *r* [m] represents the radial distance from the centroid of the charge cloud, which is assumed to move at a constant speed, *Q*(*r*,*t*) [C] represents the charge enclosed within a sphere of radius *r* at time *t*, $\mu_{e}$ [m ${}^{2}$ V ${}^{-1}$ s ${}^{-1}$ ] represents the electron mobility, *T* [K] represents temperature, $k\;=\;1.381\;\times \;10^{-23}$ m ${}^{2}$ kg s ${}^{-2}$ K ${}^{-1}$ is Boltzmann’s constant, *ε* represents the permittivity, and $q_{-}\;=\;1.602\;\times \;10^{-19}$ C is the elementary charge. Equation (41) follows directly from the continuity equation, Gauss’ Law, and Einstein’s relation for diffusion.

We integrated Eq. (41) numerically from *t* = 0 to $t\;=\;t_{\mathrm{drift}}$ , where $t_{\mathrm{drift}}$ is the drift time of the charge cloud. Since the majority of x‐ray interactions occur near the entrance of the x‐ray converter, we used $t_{\mathrm{drift}}\;=\;L^{2}/\left(\mu_{e}V\right)$ where *L* and *V* represent the converter thickness [m] and bias voltage [V], respectively. We assumed the initial charge cloud was uniform with radius $r_{0}\left(E\right)\;=\;r_{0}\cdot {\left(E/E_{0}\right)}^{1/3}$ . We used $r_{0}\;=\;17$ *μ*m and $E_{0}\;=\;60$ keV, as measured by Veale et al.;^59^ the dependence on ${\left(E/E_{0}\right)}^{1/3}$ was suggested by Taguchi et al.^35^ The charge density was calculated from $Q(r,t_{\mathrm{drift}})$ using the relation $\rho \;=\;{\left(4\pi r^{2}\right)}^{-1}\left(\partial Q/\partial r\right).$ The radial charge density (at $t\;=\;t_{\mathrm{drift}}$ ) was interpolated onto a three‐dimensional volume and then integrated numerically along the dimension perpendicular to the image plane to yield the charge‐sharing kernel.

#### 3.1.3 Calibration of the charge‐sharing model

We calibrated $p_{\mathrm{CS}}\left(r\right)$ using Redlen Technologies’ CZT bonded to a PIXIE application specific integrating circuit (ASIC).^60^, ^61^ Electron and hole mobilities for Redlen’s CZT are ∼10 ${}^{3}$ cm ${}^{2}$ V ${}^{-1}$ s ${}^{-1}$ and ∼10 ${}^{2}$ cm ${}^{2}$ V ${}^{-1}$ s ${}^{-1}$ , respectively, producing near‐unity charge collection efficiency.^58^ The experimental data used in this work were described by Thomas et al.,^58^ and were acquired using 3 × 3 arrays with pitches of 250 and 500 μm with fill factors of 0.8 and 0.7, respectively. The CZT was 2 mm in thickness and was operated at 900 V with electrons drifting toward the exit side. The energy response for each pitch was measured using americium‐241 (Am‐241) and cobalt‐57 (Co‐57) sources. Americium‐241 has a principal emission at 59.5 keV; Co‐57 has principle emissions at 122.1 and 136.5 keV. The number of counts in 0.5‐keV energy bins were recorded for each element of each array for each source. Only the spectrum from the central pixel was used for calibration.

For the Am‐241 measurements, we fit our model for path A of the energy response to the measured energy responses for energies greater than 40 keV. We implemented nonlinear least squares to determine fit parameters for $\mu_{e}$ , $\sigma_{e}$ , and $t_{\mathrm{drift}}$ . We let $t_{\mathrm{drift}}$ be a fitting parameter to account for the presence of nonuniform electric fields and boundary effects not accounted for in our model. Fit parameters were also extracted from the 136.5‐keV peak of the Co‐57 spectrum.

For each combination of energy and pitch, fit parameters were used to calculate the radius of the charge‐sharing kernel, which was defined as the half width at half maximum. We also calculated the theoretical radius using $\mu_{e}\;=\;1000$ cm ${}^{2}$ V ${}^{-1}$ s ${}^{-1}$ and a bias voltage of 900 V. A calibration was then applied by convoluting the theoretical charge‐sharing kernel with a zero‐mean, 2D Gaussian with standard deviation equal to $\left(R_{\mathrm{em}}-R_{\mathrm{th}}\right)/\sqrt{2\ln2}$ where $R_{\mathrm{em}}$ and $R_{\mathrm{th}}$ represent empirical and theoretical radii, respectively.

#### 3.1.4 Imaging parameters

##### SPCDs

We considered SPCDs exposed to mono‐energetic photons with energies equal to the average energies of the RQA7 and RQA9 x‐ray spectra (Table 2).^62^ For each spectrum, we considered detector thicknesses that yield a quantum efficiency of 90%. We considered 100‐μm elements and 500‐μm elements for the RQA7 and RQA9 spectra, respectively. These combinations of spectra and element sizes may represent applications in chest radiography and abdominal CT, respectively.

**Table 1:** Properties of CZT detectors used for numerical implementation.

Parameter	Numerical value
Charge Mobility [cm ${}^{2}$ V ${}^{-1}$ s ${}^{-1}$ ]	1000
Relative permittivity	10.6
Applied electric field [V cm ${}^{-1}$ ]	3333
Electron‐hole pair creation energy [eV]	5
Electronic noise [keV]	2

**Table 2:** Properties of RQA‐5, RQA‐7 and RQA‐9 x‐ray spectra.^62^ Also shown are the cadmium zinc telluride (CZT) converter thicknesses that yield quantum efficiencies of 70 % and 90 %. Calculations assume a density of 6.2 g cm ${}^{-3}$ .

	RQA‐5	RQA‐7	RQA‐9
Tube voltage (kV)	70	90	120
Al Filtration (mm)	21	30	40
Al half‐value layer (mm)	7.1	9.1	10.15
Average energy (keV)	52	63	76
$L_{70}$ (*μ*m)	200	341	554
$L_{90}$ (*μ*m)	430	732	1275

We also considered poly‐energetic x‐ray spectra for the combinations of spectra and element sizes listed in Table 3. X‐ray spectra were simulated using the algorithm described by Tucker et al.^63^ For each spectrum, the energy‐dependent gain and NPS were calculated for energies ranging from 10 keV to the maximum photon energy in increments of 1 keV. The resulting gains and noise power spectra were weighted by the x‐ray spectra and summed over the energy domain.

**Table 3:** Combinations of x‐ray spectra and element sizes for which the large‐area gain and digital NPS were calculated for poly‐energetic analysis of SPCD and spectroscopic x‐ray detectors.

Application	Spectrum	Element width
Angiography	RQA5	250 μm
Chest radiography I	RQA7	100 μm
Chest radiography II	RQA7	250 μm
Computed tomography	RQA9	500 μm

##### SXDs: Energy‐bin noise power spectra

We considered spectroscopic x‐ray detectors that count photons in two energy bins and calculated the NPS of each energy bin in addition to the cross NPS between energy bins. We performed calculations for the combinations of x‐ray spectra and detector element sizes in Table 3. For each combination of energy spectrum and element size, the energy threshold separating the energy bins was chosen such that approximately equal numbers of photons were recorded in each energy bin.

##### SXDs: Spectral NPS

We calculated the NPS of spectral images $\left(\tilde{S}\left(r\right)\right)$ produced by log‐subtraction of energy‐bin images:

(42) $$\tilde{S}\left(r\right)=-\log\frac{{\tilde{c}}_{L}\left(r\right)}{c_{L,0}}-\lambda \log\frac{{\tilde{c}}_{H}\left(r\right)}{c_{H,0}}$$

where ${\tilde{c}}_{L}\left(r\right)$ and ${\tilde{c}}_{H}\left(r\right)$ represent LE and HE images, and $c_{L,0}$ and $c_{H,0}$ represent reference images used for log‐normalization. The parameter *λ* is a tissue‐suppression parameter, that is, its value determines which material is suppressed from the spectral image. We considered *λ* values that suppress bone, in which case $\lambda \;=\;\mu_{L,B}/\mu_{H,B}$ where $\mu_{L,B}$ [cm ${}^{-1}$ ] represents the linear attenuation coefficient of bone averaged over the energy spectrum weighted by the energy‐dependent response of the LE bin, and similarly for $\mu_{H,B}$ .^64^, ^65^ The presampling spectral NPS was calculated as^52^, ^66^, ^67^

(43) $$W_{S}\left(u\right)=\frac{W_{L}\left(u\right)}{{\bar{c}}_{L}^{2}}+\lambda^{2}\frac{W_{H}\left(u\right)}{{\bar{c}}_{H}^{2}}+2\lambda \frac{W_{L,H}\left(u\right)}{{\bar{c}}_{L}{\bar{c}}_{H}}$$

where $W_{L}\left(u\right)$ and $W_{H}\left(u\right)$ represent the presampling noise power spectra of LE and HE images, respectively, and $W_{L,H}\left(u\right)$ represents the presampling cross NPS between LE and HE images. The digital spectral NPS was calculated using Eq. (8) for each combination of x‐ray spectrum and element size listed in Table 3.

#### 3.1.5 Spectral generalized DQE

We used the spectral NPS in Eq. (43) to calculate the zero‐frequency generalized detective quantum efficiency (GDQE) of the corresponding spectral image. Using the approach described by Richard et al.,^66^ GDQE(0) is given by:

(44) $$\text{GDQE}\left(0\right)=\frac{1+\lambda^{2}}{{\bar{q}}_{0}W_{S,\mathrm{dig}}\left(0\right)}$$

where $W_{S,\mathrm{dig}}\left(u\right)$ represents the digital NPS of the spectral image. We normalized GDQE by that of an ideal SXD that counts every incident photon in the correct energy bin. In the special case where photons are distributed equally across two energy bins, this leads to $W_{S,\mathrm{dig}}\left(0\right)\;=\;2\left(1\;+\;\lambda^{2}\right)/{\bar{q}}_{0}$ , yielding ${\text{GDQE}}_{\mathrm{ideal}}\left(0\right)\;=\;0.5$ . For a given tissue‐suppression parameter and set of energy thresholds, normalizing GDQE(0) by ${\text{GDQE}}_{\mathrm{ideal}}\left(0\right)$ yields an efficiency ranging from 0 to 1. In general, charge sharing and other stochastic image‐forming process will result in $\text{GDQE}\left(0\right)\leq {\text{GDQE}}_{\mathrm{ideal}}\left(0\right)$ .

### 3.2 Imaging simulations

We performed four sets of simulations to validate different aspects of the mathematical framework described above. The physical processes included in each set of simulations are summarized in Table 4.

**Table 4:** Summary of the different simulations used to verify our mathematical methods. The table lists some of the physical processes included in the different simulations. “Charge Cloud” refers to the finite range of photoelectrons and the expansion of charge clouds due to Coulomb forces and diffusion.

	MC1	MC2	MC3a	MC3b
Software	MCNP	MATLAB	MATLAB	MATLAB
Fluorescence	Yes	No	Yes	Yes
Compton	Yes	No	No	No
Charge cloud	No	Yes	Yes	No

The first set of simulations (MC1) was performed using the Monte Carlo N‐Particle (MCNP) transport code (version 5, the Radiation Safety Information Computational Center or RSICC, Oak Ridge, TN). These simulations did not account for the charge‐cloud model described in Section 3.A.2 and were used to validate our simplified model of characteristic emission and reabsorption, which assumes an average emission energy. We performed three additional sets of simulations, MC2, MC3a, and MC3b, in MATLAB. MC2 accounted for the charge‐cloud model described in Section 3.A.2, but ignored fluorescence; MC2 therefore assumed all interactions follow Path A in Fig. 4. MC3a included the charge‐sharing kernel in Section 3.A.2 in addition to fluorescence. MC3b accounted for fluorescence but not the charge‐sharing model.

For each type of simulation, for selected combinations of imaging parameters, we extracted the mean pixel value, standard deviation of pixel values, and ensemble NPS from multiple simulated flat‐field images. Simulation methodologies are described below.

#### 3.2.1 MCNP simulations

We used MCNP to simulate “flood field” SPCD images. Cadmium telluride detectors were modeled as 20 × 20 × *L* mm ${}^{3}$ parallelepipeds, where *L* represents thickness. We performed simulations for 63 and 76‐keV photons and thicknesses that yield a quantum efficiency of 90%. For the 63‐keV photons, we considered 100 × 100‐μm ${}^{2}$ detector elements, which may represent high‐resolution chest radiography. For 76‐keV photons, we considered 500‐μm elements, which may represent CT imaging conditions. For each set of imaging parameters, we simulated 100 images using 10 ${}^{7}$ photons/image.

To simulate photon transport and the resulting deposited energy, we used the particle‐tracking function (pTrac), which provides a report on the entire interaction history for each simulated photon.^22^, ^68^, ^69^ For each interaction event, pTrac records the interaction type, the location of the interaction, (e.g., $x_{j}^{i}$ for the *j*‐th interaction of the *i*‐th x‐ray photon transport in a simulation) and the energy remaining after the interaction (e.g., $\varepsilon_{j}^{i}$ ). To validate our model of transport and reabsorption of fluorescent photons, we assumed photons deposit all of their energy at the interaction sites, that is, we ignored the range of photoelectrons. We assumed that fluorescent x rays deposit all their energy at sites of PE reabsorption.

To produce a flat‐field SPCD image, we calculated the energy absorbed at each interaction site by subtracting the energy recorded at the previous site from that recorded at the present site (i.e., $▵E\left(x_{j}^{i}\right)\;=\;\varepsilon_{j-1}^{i}-\varepsilon_{j}^{i}$ ). The total energy deposited by the *i*‐th x‐ray photon in the *k*‐th detector element ( $v_{k}$ ) was then given by $▵E_{k}^{i}\;=\;\sum_{x\in v_{k}}▵E\left(x_{j}^{i}\right)$ . A count was incremented in element *k* when $▵E_{k}^{i}$ exceeded the predefined energy threshold.

#### 3.2.2 MATLAB simulations

Our MATLAB‐based simulations are described in detail in the supplementary material. In all cases, we assumed Poisson conversion gain and used the charge‐sharing kernel in Section 3.A.2 to randomly relocate each simulated secondary quantum relative to the site of its generation. These simulations used the same charge‐cloud model as our analytic methods.

We simulated 100 mono‐energetic flat‐field images for Chest Radiography I and Computed Tomography imaging conditions (see Table 2). For each image, we simulated 10 ${}^{5}$ photons uniformly distributed over a 100 × 100 grid of detector elements with unity fill factor. We also simulated 1000 LE and HE images of a 2‐bin SXD for Chest Radiography I (CRI) and CT conditions. For the SXD images, we simulated 3.6 × 10 ${}^{4}$ histories. The simulated LE and HE images were combined using Eq. (42) to produce a spectral image. The reference signals used for log subtraction, that is, $c_{L,0}$ and $c_{H,0}$ , were the respective mean values.

#### 3.2.3 Pixel SNR

For each set of SPCD and SXD images produced from each type of simulation, we calculated the mean pixel SNR and standard error of the pixel SNR. When calculating SNR, we only included pixels that were at least three pixels from the edges of the images. To test the prediction of Eq. (10), we fit a curve of the form $\text{SNR}\;=\;\eta \mu^{\gamma }$ where *μ* represents the mean pixel value (and *η* and *γ* are fit parameters) to the aggregated SNR data. Our theory predicts *η* = 1 and *γ* = 1/2; fit parameters within error of theoretical values and a reduced chi‐squared value ( $\chi_{\nu }^{2}$ ) close to unity support the hypothesis that pixel values are Poisson distributed.

#### 3.2.4 NPS estimation

We calculated the NPS of each image as the squared magnitude of the 2D discrete Fourier transform of the mean‐subtracted ROI with appropriate normalization.^70^ Fourier transforms were computed using the fast Fourier transform algorithm implemented in MATLAB. The resulting set of noise power spectra for each set of parameters were averaged to yield an estimate of the ensemble two‐dimensional (2D) NPS. To reduce noise, 2D noise power spectra were averaged radially.

## 4 Results

### 4.1 Charge‐sharing Kernel

**Figure 5:**
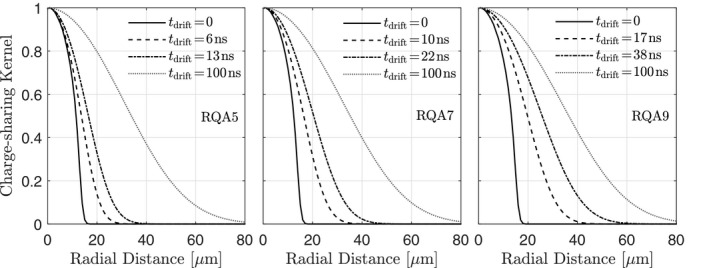
Charge‐sharing kernels [calculated by integration of Eq. (41)] for the average energies of the RQA‐series x‐ray spectra for detector thicknesses corresponding to quantum efficiencies of 70% and 90%, respectively. Also shown are charge‐sharing kernels for a 100 ns drift time.

Uncalibrated charge‐sharing kernels are shown in Fig. 5 for the average energies of the RQA5, RQA7, and RQA9 x‐ray spectra. Results are shown for detector thicknesses corresponding to quantum efficiencies of 70% and 90%. The charge‐sharing kernels are broader for higher energies because higher photon energies require thicker converters for a fixed quantum efficiency. The radius of the theoretical charge‐sharing kernel varies from ∼18 μm for an RQA5 spectrum and 70% quantum efficiency to ∼25 μm for an RQA9 spectrum and 90% quantum efficiency.

**Figure 6:**
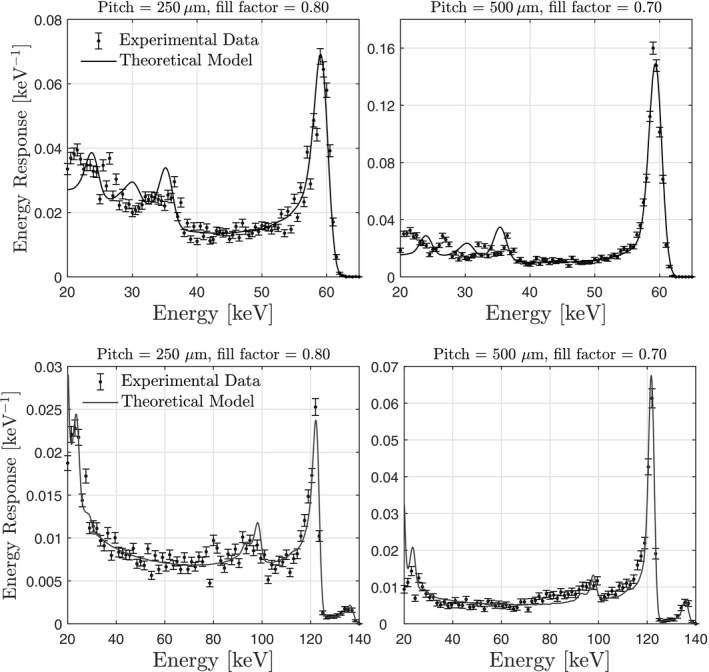
Theoretical and experimental energy response of cadmium zinc telluride chip bonded to the PIXIE application specific integrating circuit. Fit parameters used in the theoretical curves are reported in Table 5.

Figure 6 shows the energy response of Redlen’s CZT bonded to a PIXIE ASIC; also shown are energy response functions calculated from the fit parameters in Table 5. The average *μ*‐value across all four measurements is 988 ± 89 cm ${}^{2}$ V ${}^{-1}$ s ${}^{-1}$ , which is within error of the value reported by Thomas et al.^58^ However, the empirical drift time is more than twice that expected based on the mobility, bias voltage, and thickness. Based on these results, when calculating large‐area gains and noise power spectra, we convolved the nominal charge‐sharing kernels with a 2D Gaussian with standard deviation of 8 μm.

**Table 5:** Fit parameters and corresponding empirical radii ( $R_{\mathrm{em}}$ ) of the charge‐sharing kernel. Also shown are theoretical radii ( $R_{\mathrm{th}}$ ) of the charge‐sharing kernels.

	59 keV		136 keV	
	250 μm	500 μm	250 μm	500 μm
*μ* [cm ${}^{2}$ /(V·s)]	996 ± 13	867 ± 15	983 ± 48	1040 ± 72
$\sigma_{e}$ [keV]	0.64 ± 0.05	0.56 ± 0.04	0.86 ± 0.46	0.66 ± 0.50
$t_{\mathrm{drift}}$ [ns]	100 ± 1	115 ± 1	102 ± 1	96 ± 1
$R_{\mathrm{em}}$ [*μ*m]	35	35	42	42
$R_{\mathrm{th}}$ [*μ*m]	26		32	

There is reasonable agreement between theoretical and experimental energy response functions. Discrepancies between theory and experiment occur near the reabsorption and escape peaks. This is due to the use of an average characteristic emission energy in the theoretical calculations. This discrepancy is expected to have a negligible effect in systems designed for imaging applications, for which the electronic noise level is ∼2 keV, which will blur the $K_{\alpha }$ and $K_{\beta }$ lines together.

### 4.2 Pixel SNR

**Figure 7:**
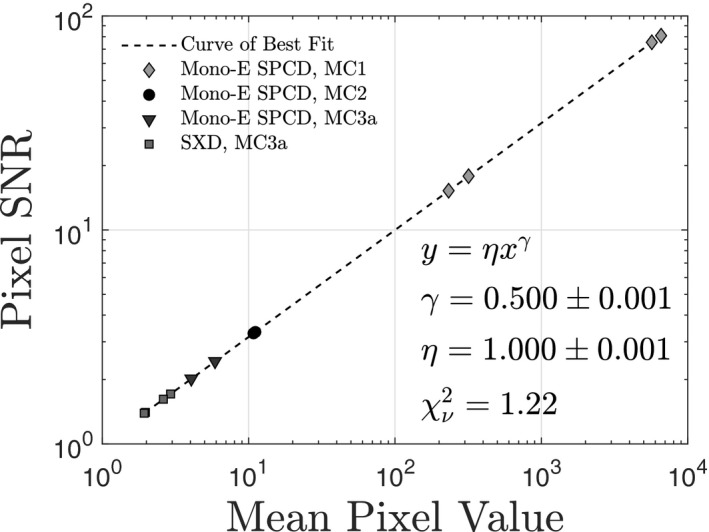
Pixel signal‐to‐noise ratio vs. mean pixel value for simulated images. Error bars are not shown because they are much smaller than the symbol sizes. Also shown is the curve of best fit, which shows that pixel values are Poisson distributed.

Figure 7 shows pixel SNRs extracted from simulated images as a function of mean pixel value. Also shown is the curve of best fit to the aggregated mono‐energetic SPCD, poly‐energetic SPCD and SXD data. Fit parameters are within error of those predicted by Eq. (10). This agreement, together with the $\chi_{\nu }^{2}$ value close to unity, supports the prediction that pixel values are Poisson distributed, despite the presence of charge sharing.

### 4.3 Charge sharing and noise aliasing in SPCDs

**Figure 8:**
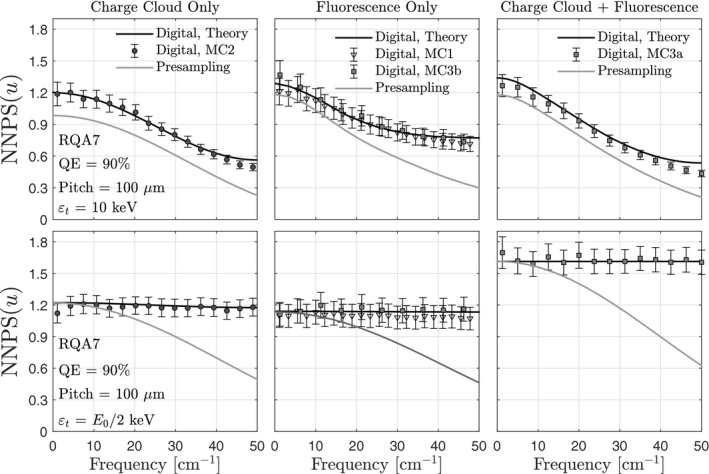
Presampling and digital NNPS for the average energy of the RQA7 x‐ray spectrum, 100 × 100‐μm ${}^{2}$ elements, and a converter thickness that yields a quantum efficiency of 90%; $\varepsilon_{t}$ represents the energy threshold. Also shown are the results of MC simulations.

**Figure 9:**
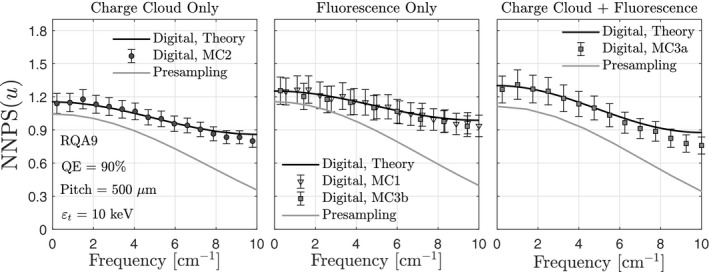
Presampling and digital NNPS for the average energy of the RQA9 x‐ray spectrum, 500 × 500‐μm ${}^{2}$ elements, and a converter thickness that yields a quantum efficiency of 90%; $\varepsilon_{t}$ represents the energy threshold. Also shown are the results of MC simulations.

Figure 8 shows noise power spectra for CRI imaging conditions with and without fluorescence, and with and without the charge cloud. Also shown are the results of simulations MC1, MC2, MC3a, and MC3b. In all cases, there is good agreement between theory and MC simulations. There is also good agreement between MC3b and MC1, demonstrating that the MATLAB‐based simulations of PE interactions (without the charge cloud) accurately predict those of MCNP. The top row of images in Fig. 8 shows results for a threshold equal to the electronic noise floor, which was assumed to be 10 keV. Differences between the presampling and digital NNPS are due to noise aliasing, which is substantial at zero frequency for both CRI and CT imaging conditions, the latter of which is illustrated in Fig. 9. Increasing the energy threshold to half of the incident photon energy (bottom row of Fig. 8) removes zero‐frequency noise aliasing, but decreases the number of detected photons, increasing the presampling NPS at zero frequency and increasing the digital NNPS near the Nyquist frequency.

**Figure 10:**
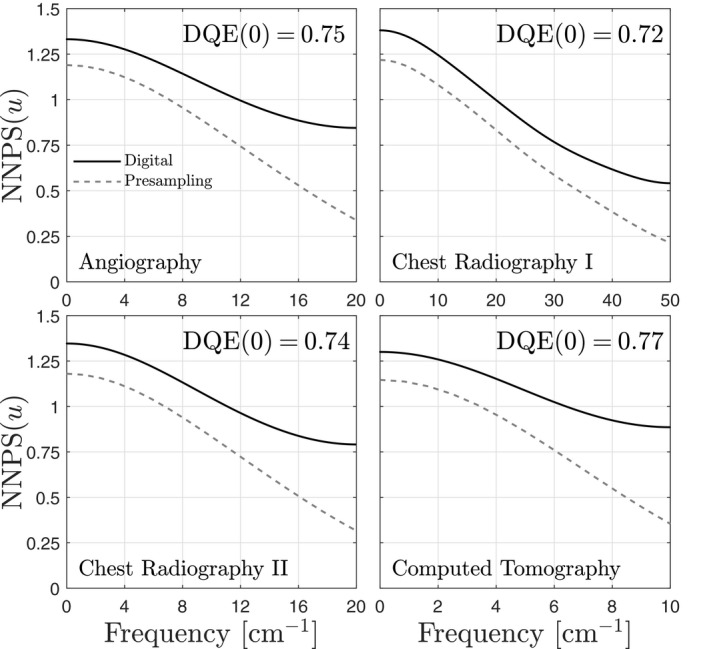
Presampling and digital SPCD NNPS for selected applications. Imaging parameters for each application are listed in Table 3. Results are plotted up to the Nyquist frequency.

Poly‐energetic SPCD noise power spectra are illustrated in Fig. 10. Similar to the mono‐energetic results, there is noise aliasing across all spatial frequencies, including zero frequency. Noise aliasing near the Nyquist frequency decreases for smaller element sizes due to greater sharing of charge between elements.

### 4.4 Zero‐frequency DQE of SPCDs

Zero‐frequency DQEs of SPCDs are shown in Fig. 10. For each case, the quantum efficiency is 90%, but DQE(0) varies from 0.74 to 0.77 depending on the combination of energy spectrum and element size. These results suggest that charge sharing due to the finite range of photoelectrons, the expansion of charge clouds, and reabsorption of characteristic photons may reduce DQE(0) by 13% to 18% depending on the application.

**Figure 11:**
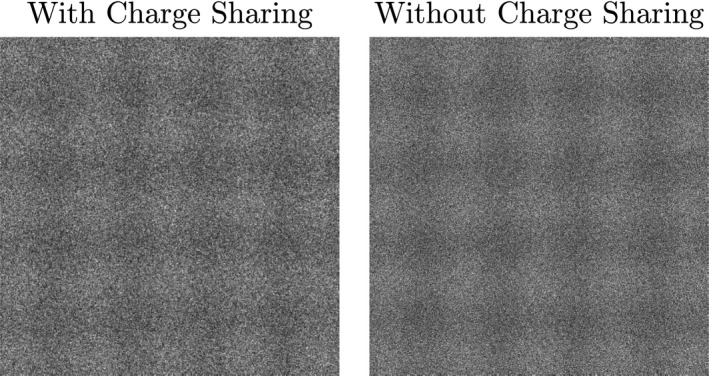
Simulated images of a two‐dimensional cosine function (spatial frequency  = 1 cycle/cm) with and without charge sharing. Images with and without charge sharing are simulated with the same pixel SNR. Images were simulated using an RQA7 x‐ray spectrum, 100‐μm elements, and a 732‐μm thick x‐ray converter.

The effect of low‐frequency noise aliasing on signal detection is illustrated visually in Fig. 11, which shows simulated images of a 2D cosine with a spatial frequency of 1 cycle/cm. The images with and without charge sharing have the same pixel SNR, and the MTFs with and without charge sharing are approximately equal at 1 cycle/cm^31^; differences in signal visibility are primarily attributable to differences in noise texture.

### 4.5 Charge sharing and noise aliasing in SXDs

**Figure 12:**
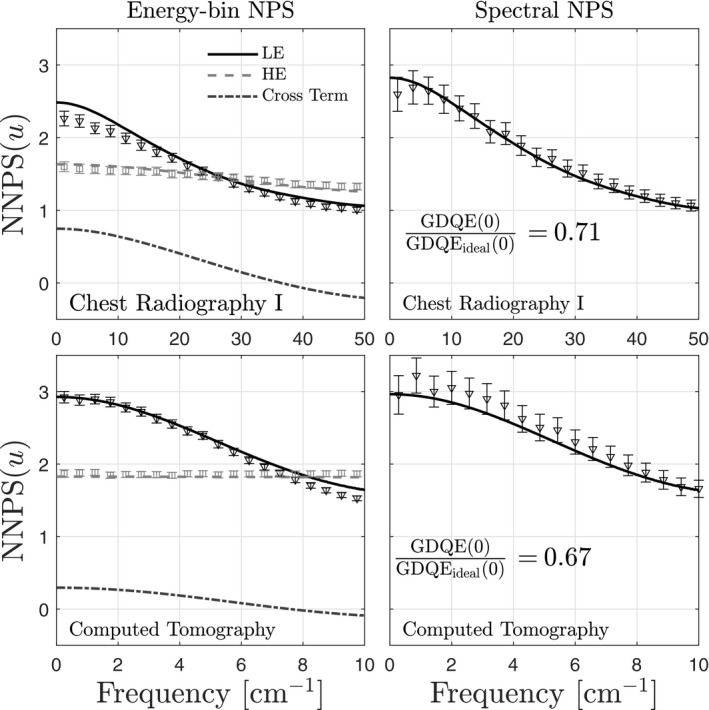
Energy‐bin noise power spectra and corresponding spectral noise power spectra for chest radiography and computed tomography imaging conditions. Symbols represent results from MC3a simulations. Results are shown for tissue‐suppression parameters that suppress bone. Also show is the generalized zero‐frequency detective quantum efficiency.

Energy‐bin noise power spectra are shown in the left column of Fig. 12 for CRI and CT imaging conditions. There is reasonable agreement between theory and simulation. In general, the LE digital NNPS is highly correlated, dropping by 60% and 50% from zero frequency to the Nyquist frequency for CRI and CT, respectively. In contrast, noise in the HE images is approximately uncorrelated. This difference between LE and HE images is caused by charge sharing, which produces LE deposition events in elements neighboring those of primary interactions; these events are recorded primarily in the LE bin. In contrast, for the energy thresholds used in this study, the HE bin is only sensitive to events counted in elements in which primary interactions occur.

Figure 12 also shows cross noise power spectra of LE and HE images; these cross terms quantify spatio‐energetic noise correlations and are non‐negligible. It is noteworthy that the cross NPS is negative near the Nyquist frequency. Mathematically, the cross NPS must be negative somewhere within the Nyquist region because the cross NPS must integrate to zero, as predicted by Eq. (5). Physically, a negative cross NPS near the Nyquist frequency means that an increase in LE counts in an element is related to a decrease in HE counts in a neighboring element, which likely occurs when sharing of energy between two elements leads to multiple counts in LE bins.

### 4.6 Spectral NPS and zero‐frequency GDQE

The spectral NPS is shown in the right column of Fig. 12 for CRI and CT imaging conditions. Also shown are the results of MC3a; there is reasonable agreement between theory and MC. In general, the spectral NPS is highly correlated, primarily because of the highly correlated LE NPS. Also shown in Fig. 12 is the zero‐frequency GDQE normalized by that of an ideal spectral image formed using equal distribution of photons across two energy bins. Reductions in GDQE(0) are similar in magnitude, but slightly greater, than DQE(0) reductions for SPCDs.

## 5 Discussion

We have presented new mathematical methods for modeling the NPS of SPCDs and the NPS of spectral images obtained from SXDs. While we only considered SXDs with two energy bins, the methods developed here also apply to systems that use three or more energy bins. We used these methods to analyze noise correlations in SPCDs, the zero‐frequency DQE of SPCDs, and the zero‐frequency GDQE of SXDs.

We showed that the autocovariance and NPS can be calculated from knowledge of the joint PDF of deposited energies, which describes the probability of recording two photons of two different energies in two different elements following a single x‐ray interaction. This joint PDF of deposited energies is a presampling, prethresholding metric, describing presampling noise correlations in both the spatial and energy domains. Equations (3), (4), (5), (6), (7), (8), (9) combine to make a very important point. They show that the joint PDF in Eq. (3) determines the presampling cross covariance, which can in turn be used to determine the presampling NPS and cross NPS, in addition to the digital NPS and digital cross NPS. This differs from some early investigations in which energy deposition was calculated in neighboring elements and then discrete element signals were used to determine interelement covariances for two reasons. The first is that integrating detected photons over an element area does not preserve the presampling covariance and introduces an error by averaging over the element width. The second is that use of discrete values in the covariance calculation incorporates sampling and aliasing errors that do not correctly represent the digital covariance. Integrating photon counts in an element and then calculating element correlations is not the same as averaging element correlations that result from interactions at random positions in the elements.

The firm theoretical foundation of Eqs. (3), (4), (5), (6), (7), (8), (9) shows that the correct way to determine the NPS is from a determination of the energy deposition joint PDF. This relationship between NPS and joint PDF of deposited energies assumes an LSI imaging system, but is otherwise generic and independent of the methodology used calculate the joint PDF. We calculated it analytically, but it could also be calculated by MC‐based approaches. Alternatively, it may be practical to measure the joint PDF experimentally by scanning a thin (much smaller than the element width) pencil beam of radiation across a detector. The joint PDF formalism applies to both SPCDs and SXDs, thus providing a unified framework for theoretical analysis of frequency‐dependent noise in photon‐counting x‐ray imaging.

We also showed that the number of detected photons per element of an SPCD or SXD remains Poisson distributed in the presence of charge sharing. Poisson‐distributed counts was predicted by Stierstorfer et al.,^32^ demonstrated experimentally by Ji et al.,^71^ and assumed by Michel et al.^37^, ^38^ in their multiplicity analysis. What we have shown is that charge sharing with a multiplicity greater than unity must increase pixel SNR, but, in doing so, shifts high‐frequency noise to low frequencies, including zero frequency. In all cases considered, this effect reduced DQE(0) and GDQE(0) by greater than 10%, even for larger elements (i.e., 500 μm) used in CT applications. This reduction in zero‐frequency performance is similar in magnitude to Swank noise in energy‐integrating systems,^72^ suggesting that, in cases where electronic noise is negligible but charge sharing is not, SPCDs may offer no advantage over EIDs in terms of zero‐frequency signal‐to‐noise performance.

Additionally, we showed that for the special case of zero‐frequency analysis of systems with a single, open energy bin, the joint PDF framework yields a DQE(0) value equivalent to that predicted from the multiplicity approach.^37^, ^38^ Our approach therefore generalizes the multiplicity framework to nonzero frequencies and to systems with multiple energy bins.

Comparison of the presampling NPS with the digital NPS showed that it is actually the combination of charge sharing and noise aliasing that is responsible for shifting of high‐frequency noise to low frequencies. This analysis highlights the utility of modeling both the presampling and digital NPS, the former of which is not typically accessible by experiment. Analysis of presampling image noise is commonplace in cascaded systems analysis of energy‐integrating systems, but has been overlooked for photon‐counting systems, likely because most efforts to model photon‐counting systems have been based on MC methods. While MC methods, for example implemented in MCNP or Geant, provide highly accurate physical models, it is difficult to turn on and off different physical process, for example, fluorescence, and to incorporate charge transport. In contrast, our approach enables interrogating separately and together the effects of x‐ray fluorescence, the size of charge clouds, and sampling. This theoretical approach is congruous with historical efforts to model and understand the imaging performance of energy‐integrating x‐ray detectors.

While our experimental configuration (i.e., a 3 × 3 grid of elements) prohibited direct comparison of theoretical and experimental noise power spectra, we validated our model of the production, transport, and reabsorption of fluorescence x rays against MC simulations performed using MCNP, and calibrated our charge‐cloud model against empirical data. These validations support the accuracy of our models, but our models are still idealized. For example, our model does not account for incomplete charge collection, the small‐pixel effect, and threshold dispersion. While charge mobility in modern CZT and CdTe is high enough to yield near‐unity charge collection when there is sufficient time for charge collection,^57^, ^58^, ^73^, ^74^, ^75^ exploiting the small pixel effect in high‐count rate applications may reduce collection efficiencies.^76^ In addition, threshold dispersion may have a non‐negligible effect on image noise. As such, the models presented here represent upper limits of system performance, serving as benchmarks against which actual detector performance can be compared.

Our charge‐cloud model was derived from charge transport theory and accounts for the initial sizes of charge clouds, diffusion of charges, and Coulomb repulsion of charges of like sign. Our approach enables calculating the charge cloud as function of photon energy, detector thickness, mobility of charge carriers, and the potential difference applied across the x‐ray converter. The calibration required to achieve agreement between our model and experiments was approximately independent of photon energy and pixel pitch, suggesting that our model accurately describes the energy dependence and pitch dependence of the charge‐sharing tail.

Our x‐ray interaction model did not account for reabsorption of Compton‐scattered x rays. While Compton scattering accounts for less than 5% of interactions in CdTe and CZT for the RQA5, RQA7, and RQA9 x‐ray spectra, this will not be the case for silicon‐based systems^77^ currently under development. Modeling of systems that use a silicon x‐ray converter will require extension of our model to include Compton scattering, for example using an approach similar to Yun et al.^22^

A substantial limitation of the frequency‐dependent model presented here, and those of other groups employing MC‐based approaches, is the omission of pulse pile‐up. Pulse pile‐up produces a nonlinear relationship between input and output count rates. While the statistical variance of the number of detected photons in the presence of pulse pile‐up has been described,^78^ frequency‐dependent effects have not. A frequency‐dependent description of pulse pile‐up is a nontrivial theoretical problem, and is a focus of ongoing research.

This work did not consider the frequency‐dependent DQE, which requires a model of the MTF. Incorporation of the NPS framework developed here into analyses of the frequency‐dependent DQE of SPCDs and GDQE of SXDs will be a focus of future work.

## 6 Conclusions

We present a new analysis of the NPS of photon‐counting x‐ray detectors, including those that use multiple thresholds to estimate the spectrum of interacting photon energies. The analysis and mathematical methods enable theoretical modeling and understanding of image noise in photon‐counting x‐ray imaging. Specific conclusions from this work consist of the following points.

1. The energy deposition PDF $p_{\varepsilon }\left(\varepsilon ;r\right)$ (when integrated over the energy domain) represents the point‐spread function of photon‐counting energy‐resolving x‐ray detectors. Its Fourier transform therefore gives the presampling MTF. Combined with the element sampling frequency and frequency‐aliasing considerations, this gives a comprehensive description of detector performance within the limits of assumptions made (linearity, shift invariance, no pulse pile‐up).
2. Noise performance, including spatial and energy correlations between elements and energy bins, is described by the joint PDF of deposited energies which provides a method of determining the presampling cross covariance, Eq. (3), and Wiener NPS, Eq. (6), and cross NPS, Eq. (8), in spectral imaging, including noise‐aliasing effects. The joint PDF can be determined in any way, including the theoretical cascaded systems analysis used here, by Monte Carlo study, or by direct experimental measurement using a scanning x‐ray beam.
3. Charge sharing, when left uncorrected, causes zero‐frequency noise aliasing that reduces the zero‐frequency performance of SPCDs and SXDs. Methods developed here can be incorporated into task‐based assessment of image quality, and will be useful in the design and optimization of novel applications of photon‐counting x‐ray imaging technology.
4. Theoretical models of charge sharing and fluorescence reabsorption are all validated by a Monte Carlo study and experimentally for a CZT/CdTe‐based energy‐resolving photon‐counting detector.

## Conflict of interest

Dr. Iniewski is the Manager of Research and Development at Redlen Technologies.

## Supporting information


**Data S1:** Supplemental material.

## Appendix A List of Parameters

Table 6 lists selected parameters used in this work.

**Table 6:** List of parameters.

Symbol	Description	Units
${\bar{q}}_{0}$	Fluence of x‐ray quanta incident on detector	mm ${}^{-2}$
${\bar{N}}_{0}$	Total number of x‐ray quanta incident on detector	unitless
*a*	Detector element area	mm ${}^{2}$
*A*	Detector area	mm ${}^{2}$
**r**	2D vector in the spatial domain	mm
$r_{0}$	Location of primary x‐ray incidence	mm
**u**	2D vector in the frequency domain	mm ${}^{-1}$
***τ***	2D vector in the spatial domain	mm
$\tilde{d}\left(r\right)$	Pre‐sampling detector signal	
${\bar{c}}_{i}$	Average number of photons detected in energy *j*	unitless
$K_{i,j}\left(\tau \right)$	Pre‐sampling autocovariance between energy bins *i* and *j*	unitless
$W_{i,j}\left(u\right)$	Pre‐sampling cross noise power spectrum for energy bins *i* and *j*	mm ${}^{2}$
$W_{\mathrm{dig},i,j}\left(u\right)$	Digital cross noise power spectrum for energy bins *i* and *j*	mm ${}^{2}$
*E*	Energy of incident x‐ray quanta	keV
*ɛ*	Deposited photon energy	keV
$p_{\varepsilon }\left(\varepsilon ;r\right)$	PDF of *ɛ* for an element lefted at **r** relative to an x‐ray interaction	keV ${}^{-1}$
$p_{\varepsilon ,X}\left(\varepsilon \right)$	PDF of *ɛ* for an element centered at **r** relative to an x‐ray interaction given a photon that follows path *X*	keV ${}^{-1}$
$p_{\varepsilon ,\varepsilon^{\prime }}\left(\varepsilon ,\varepsilon^{\prime };\tau \right)$	Joint PDF of deposited energies for elements separated by ***τ***	keV ${}^{-2}$
${\bar{\varepsilon }}_{X}\left(r\right)$	Average energy deposited in an element centered at **r** relative to primary interaction for path *X*	keV
$\sigma_{X}^{2}\left(r\right)$	Variance of energy deposited in an element centered at **r** relative to primary interaction for path *X*	keV ${}^{2}$
$N_{X}\left(\varepsilon ,r\right)$	Normal distribution describing the PDF of *ɛ* for path *X*	keV ${}^{-1}$
$p_{\mathrm{CS}}\left(r\right)$	Charge‐sharing kernel accounting for the width of the charge clouds	mm ${}^{-2}$
$\Pi \left(\frac{r}{a}\right)$	2D rectangle function of area *a*	unitless
$\sigma_{e}$	Electronic noise level	keV
$p_{K}\left(r\right)$	K‐shell reabsorption kernel, equal to the PDF of reabsorbing a fluorescent photon at **r** relative to the site of generation	mm ${}^{-2}$
$P_{K}$	K‐shell participation fraction	unitless
$\omega_{K}$	K‐shell fluorescence yield	unitless
$l_{j}$	Low‐energy threshold for energy bin *j*	keV
$u_{j}$	High‐energy threshold for energy bin *i*	keV
$\mu_{e}$	Electron mobility	cm ${}^{2}$ /V
*ε*	Permittivity	F/m
$\xi_{\mathrm{PE}}$	Probability of photo‐electric (PE) interaction given an interaction	unitless
$\nu_{\mathrm{Cd}}$	Probability of interaction in Cd given an interaction in CdTe	unitless
$\nu_{\mathrm{Te}}$	Probability of interaction in Te given an interaction in CdTe	unitless
$f_{K}$	probability of reabsorption for K‐shell photons	unitless
$L_{70}$	Detector thickness required to yield a quantum efficiency of 70%	mm
$L_{90}$	Detector thickness required to yield a quantum efficiency of 90%	mm

## Appendix B Autocovariance of the Photon‐counting Image Signal

We let ${\tilde{s}}_{i}^{j}\left(r\right)$ represent the presampling, thresholded image signal for energy bin *j* and photon *i*. The presampling image for energy bin *i* is then

(B1) $${\tilde{c}}_{i}^{†}\left(r\right)=\sum_{i=1}^{{\tilde{N}}_{0}}{\tilde{s}}_{j}^{i}\left(r\right)$$

where ${\tilde{N}}_{0}$ represents the number of incident quanta. The presampling covariance between energy bins *i* and $i^{\prime }$ of elements separated by ***τ*** is given by

(B2) $$K_{j,j^{\prime }}\left(\tau \right)=R_{j,j^{\prime }}\left(r,r+\tau \right)-{\bar{c}}_{j}{\bar{c}}_{j^{\prime }}$$

where $R_{j,j^{\prime }}\left(r,r\;+\;\tau \right)$ represents the correlation between ${\tilde{c}}_{j}\left(r\right)$ and ${\tilde{c}}_{j^{\prime }}\left(r\;+\;\tau \right)$ :

(B3) $$R_{j,j^{\prime }}\left(\tau \right)=E\left[{\tilde{c}}_{j}\left(r\right){\tilde{c}}_{j^{\prime }}\left(r+\tau \right)\right]$$

(B4) $$=E\left[\sum_{i=i}^{{\tilde{N}}_{0}}\sum_{i^{\prime }=}^{{\tilde{N}}_{0}}{\tilde{s}}_{j}^{i}\left(r\right){\tilde{s}}_{j^{\prime }}^{i^{\prime }}\left(r+\tau \right)\right].$$

Separating the summation into terms for which $i\;=\;i^{\prime }$ and those for which $i\neq i^{\prime }$ :

(B5) $$\begin{array}{cc} R_{j,j^{\prime }}\left(\tau \right) & =E\left[\sum_{i=1}^{{\tilde{N}}_{0}}{\tilde{s}}_{j}^{i}\left(r\right){\tilde{s}}_{j}^{i^{\prime }}\left(r+\tau \right)\right]\\ & +E\left[\sum_{i=1}^{{\tilde{N}}_{0}}\sum_{i^{\prime }=1,i\neq i^{\prime }}^{{\tilde{N}}_{0}}{\tilde{s}}_{j}^{i}\left(r\right){\tilde{s}}_{j^{\prime }}^{i^{\prime }}\left(r+\tau \right)\right]. \end{array}$$

We first average over ${\tilde{s}}_{j}^{i}(r$ and ${\tilde{s}}_{j^{\prime }}^{i^{\prime }}\left(r\;+\;\tau \right)$ for fixed ${\tilde{N}}_{0}$ , in which case

(B6) $$E\left[\sum_{i=1}^{{\tilde{N}}_{0}}{\tilde{s}}_{j}^{i}\left(r\right){\tilde{s}}_{j}^{i^{\prime }}\left(r+\tau \right)|{\tilde{N}}_{0}\right]=\sum_{i=1}^{{\tilde{N}}_{0}}E[{\tilde{s}}_{j}^{i}\left(r\right){\tilde{s}}_{j}^{i^{\prime }}\left(r+\tau \right)]$$

and

(B7) $$E\left[\sum_{i=1}^{{\tilde{N}}_{0}}\sum_{i^{\prime }=1,i\neq i^{\prime }}^{{\tilde{N}}_{0}}{\tilde{s}}_{j}^{i}\left(r\right){\tilde{s}}_{j^{\prime }}^{i^{\prime }}\left(r+\tau \right)|{\tilde{N}}_{0}\right]$$

(B8) $$=\sum_{i=1}^{{\tilde{N}}_{0}}\sum_{i^{\prime }=1,i\neq i^{\prime }}^{{\tilde{N}}_{0}}E[{\tilde{s}}_{j}^{i}\left(r\right)]E[{\tilde{s}}_{j^{\prime }}^{i^{\prime }}\left(r+\tau \right)]$$

(B9) $$={\tilde{N}}_{0}\left({\tilde{N}}_{0}-1\right){\bar{s}}_{j}{\bar{s}}_{j^{\prime }}$$

where we have used the fact that ${\tilde{s}}_{j}^{i}\left(r\right)$ is independent of ${\tilde{s}}_{j^{\prime }}^{i^{\prime }}\left(r\;+\;\tau \right)$ for $i\neq i^{\prime }$ , and ${\bar{s}}_{j}$ represents the average number of photons counted in energy bin *j* per detector element given one incident photon. Noting that $E[{\tilde{s}}_{j}^{i}\left(r\right){\tilde{s}}_{j}^{i^{\prime }}\left(r\;+\;\tau \right)]$ is independent of *i*, and averaging over ${\tilde{N}}_{0}$ yields

(B10) $$\begin{array}{cc} R_{j,j^{\prime }}\left(\tau \right) & ={\bar{N}}_{0}E\left[{\tilde{s}}^{i}\left(r\right){\tilde{s}}^{i^{\prime }}\left(r+\tau \right)\right]\\ & +\left(\sigma_{N_{0}}^{2}+{\bar{N}}_{0}^{2}-{\bar{N}}_{0}\right){\bar{s}}_{j}{\bar{s}}_{j^{\prime }} \end{array}$$

(B11) $$={\bar{N}}_{0}E\left[{\tilde{s}}_{j}\left(r\right){\tilde{s}}_{j^{\prime }}\left(r+\tau \right)\right]+{\bar{c}}_{j}{\bar{c}}_{j^{\prime }}$$

where ${\bar{c}}_{j}\;=\;{\bar{N}}_{0}{\bar{s}}_{j}$ and we have assumed ${\tilde{N}}_{0}$ is Poisson distributed, that is, $\sigma_{N_{0}}^{2}\;=\;{\bar{N}}_{0}$ . Combining Eq. (B2) with Eq. (B11) yields

(B12) $$K_{j,j^{\prime }}\left(\tau \right)={\bar{N}}_{0}E\left[{\tilde{s}}_{j}\left(r\right){\tilde{s}}_{j}\left(r+\tau \right)\right]$$

which leads directly to Eq. (3).

## Appendix C Connection with the multiplicity framework

The multiplicity is defined as the number of detected photons per interacting photon, independent of where photons are detected. Letting ${\tilde{\eta }}_{l,n}$ represent the number of photons detected in element *l*,*n* given an interaction, the multiplicity is given by

(C1) $$\tilde{m}=\sum_{l,n=0}^{\infty }{\tilde{\eta }}_{\pm l,\pm n}.$$

The variance of the multiplicity is given by

(C2) $$\sigma_{m}^{2}=\bar{m^{2}}-{\bar{m}}^{2}=N^{2}\sum_{l,n=0}^{\infty }K_{\pm l,\pm n}^{\eta }$$

where $N^{2}$ represents the number of elements and $K_{l,n}^{\eta }$ represents the covariance between elements separated by *l* elements in the *x* and *n* elements in the *y* directions, and we have assumed WSS. The covariance is given generically by

(C3) $$K_{l,n}^{\eta }=E\left[\eta_{l^{\prime },n^{\prime }}\eta_{l^{\prime }+l,n^{\prime }+n}\right]-{\bar{\eta }}^{2}$$

where

(C4) $$E\left[\eta_{l^{\prime },n^{\prime }}\eta_{l^{\prime }+l,n^{\prime }+n}\right]=P\left(\eta_{l^{\prime },n^{\prime }}=1\;\text{AND}\;\eta_{l^{\prime }+l,n^{\prime }+n}=1\right)$$

where $P(\eta_{l^{\prime },n^{\prime }}\;=\;1\;\text{AND}\;\eta_{l^{\prime }\;+\;l,n^{\prime }\;+\;n}\;=\;1)$ represents the probability that $\eta_{l^{\prime },n^{\prime }}\;=\;1$ and $\eta_{l^{\prime }\;+\;l,n^{\prime }\;+\;n}\;=\;1$ and is the same for all $l^{\prime }$ and $n^{\prime }$ . Since $\eta_{l,n}$ represents the number of photons in element *l*,*n* given one interaction, $P(\eta_{l^{\prime },n^{\prime }}\;=\;1\;\text{AND}\;\eta_{l^{\prime }\;+\;l,n^{\prime }\;+\;n}\;=\;1)$ is related to the joint distribution of deposited energies:

(C5) $$E\left[\eta_{l^{\prime },n^{\prime }}\eta_{l^{\prime }+l,n^{\prime }+n}\right]=\frac{1}{\alpha }\int_{\varepsilon_{t}}^{\infty }\int_{\varepsilon_{t}}^{\infty }p_{\varepsilon ,\varepsilon^{\prime }}\left(\varepsilon ,\varepsilon^{\prime };\tau_{l,n}\right)d\varepsilon d\varepsilon^{\prime }$$

where $\tau_{l,n}\;=\;\left(l\Delta ,n\Delta \right)$ . The factor of 1/*α* accounts for the fact that the multiplicity is the number of photons per interaction, whereas $p_{\varepsilon ,\varepsilon^{\prime }}\left(\varepsilon ,\varepsilon^{\prime };\tau \right)$ is the joint distribution of deposited energies per incident photon. Combining Eqs. (3), (C2) and (C5), and noting that the sampled autocovariance is related to the presampling autocovariance through a factor of *a* yields

(C6) $$\bar{m^{2}}=\frac{1}{{\bar{q}}_{0}a\alpha }\sum_{l,n=0}^{\infty }K_{\pm l,\pm n}$$

which leads to Eq. (16).

## Appendix D The poisson approximation

We show here that Eqs. (19), (20), (21), (22), (23), (24), (25) correspond to *F* = 1, that is, Poisson‐distributed conversion gain, and are approximate expressions when *F*≪1 and $\bar{g}\gg 1$ . We consider the number of quanta ( $\tilde{n}$ ) collected in an element centered at **r** given $\tilde{g}$ quanta liberated at the origin. In this case, $\tilde{n}$ is binomially distributed with the following probability mass function:

(D1) $$P\left(\tilde{n}=n|\tilde{g}\right)=\left(\begin{array}{c} \tilde{g}\\ n \end{array}\right){\left[P_{\mathrm{CS}}\left(r\right)\right]}^{n}{\left[1-P_{\mathrm{CS}}\left(r\right)\right]}^{\tilde{g}-n}$$

where $P(\tilde{n}=n|\tilde{g})$ represents the probability that $\tilde{n}=n$ given $\tilde{g}$ total quanta. Averaging over $\tilde{g}$ yields

(D2) $$P\left(\tilde{n}=n\right)=\sum_{g=n}^{\infty }P\left(\tilde{n}=n|g\right)P\left(\tilde{g}=g\right)$$

where $P(\tilde{g}=g)$ represents the probability that $\tilde{g}=g$ . Note that the sum is over all *g*≥*n*.

### D.1. Special case: *F*=1

In this case, $P(\tilde{g}=g)$ is a Poisson distribution with mean $\bar{g}$ , yielding

(D3) $$P\left(\tilde{n}=n\right)=\sum_{g=n}^{\infty }\frac{g!{\bar{g}}^{g}e^{-\bar{g}}}{n!(g-n)!g!}{\left[P_{\mathrm{CS}}\left(r\right)\right]}^{n}{\left[1-P_{\mathrm{CS}}\left(r\right)\right]}^{g-n}$$

(D4) $$=\frac{e^{-\bar{g}}}{n!}{\left[P_{\mathrm{CS}}\left(r\right)\right]}^{n}\sum_{g=n}^{\infty }\frac{{\bar{g}}^{g}{\left[1-P_{\mathrm{CS}}\left(r\right)\right]}^{g-n}}{\left(g-n\right)!}.$$

After some simplifications, the preceding expression can be rewritten as:

(D5) $$P\left(\tilde{n}=n\right)=\frac{e^{-\bar{g}}}{n!}{\left[\bar{g}P_{\mathrm{CS}}\left(r\right)\right]}^{n}\sum_{k=0}^{\infty }\frac{{\bar{g}}^{k}{\left[1-P_{\mathrm{CS}}\left(r\right)\right]}^{k}}{k!}.$$

We recognize the summation as the exponential function with argument $\bar{g}[1-P_{\mathrm{CS}}\left(r\right)]$ , yielding

(D6) $$P\left(\tilde{n}=n\right)=\frac{e^{-\bar{g}P_{\mathrm{CS}}\left(r\right)}}{n!}{\left[\bar{g}P_{\mathrm{CS}}\left(r\right)\right]}^{n}$$

which is the Poisson distribution with mean $\bar{g}P_{\mathrm{CS}}\left(r\right)$ . Approximating the Poisson distribution as normal and accounting for electronic noise leads to Eqs. (19), (20), (21), (22), (23), (24), (25).

### D.2. Special case: *F*≪1

In this case, we ignore the width of the distribution of conversion gains, in which case $P\left(\tilde{g}\;=\;g\right)\approx \delta_{g\bar{g}}$ where $\delta_{g\bar{g}}$ represents the Kronecker delta function. Equation (D2) reduces to

$$P\left(\tilde{n}=n\right)=P\left(\tilde{n}=n|\bar{g}\right)$$

where $P(\tilde{n}=n|\bar{g})$ is a binomial distribution with $\bar{g}$ trials and probability of success $P_{\mathrm{CS}}\left(r\right)$ . Assuming $\bar{g}\gg 1$ and invoking the Poisson approximation to the binomial distribution leads to Eq. (D6). Approximating the Poisson distribution as Normal and accounting for electronic noise leads to Eqs. (19), (20), (21), (22), (23), (24), (25).

## Appendix E Cross covariance for paths B and C

Here, we show how to derive Eq. (36) from Eq. (33). First, we expand the convolutions in the second term of Eq. (33):

(E1) $$I=\int_{A}\left[N_{B}\left(\varepsilon ;r\right)N_{B}\left(\varepsilon^{\prime };r+\tau \right)\right]{*}_{\varepsilon ,\varepsilon^{\prime }}[p_{K}\left(r\right){*}_{r}\left[N_{C}\left(\varepsilon ;r\right)N_{C}\left(\varepsilon^{\prime };r+\tau \right)\right]]d^{2}r.$$

(E2) $$\begin{array}{cc} & =\int_{A}\int_{A}\int_{-\infty }^{+\infty }\int_{-\infty }^{+\infty }N_{B}\left(\eta -\varepsilon ;r\right)N_{B}\left(\eta^{\prime }-\varepsilon^{\prime };r+\tau \right)N_{C}\left(\eta ;r-\Delta r\right)N_{C}\left(\eta^{\prime };r+\tau -\Delta r\right)\\ & \times p_{K}\left(\Delta r\right)d\eta d\eta^{\prime }d^{2}rd^{2}\Delta r \end{array}$$

(E3) $$\begin{array}{cc} & =\int_{A}\int_{A}\left[\int_{-\infty }^{+\infty }N_{B}\left(\eta -\varepsilon ;r\right)N_{C}\left(\eta ;r-\Delta r\right)\right]d\eta \left[\int_{-\infty }^{+\infty }N_{B}\left(\eta^{\prime }-\varepsilon^{\prime };r+\tau \right)N_{C}\left(\eta^{\prime };r+\tau -\Delta r\right)d\eta^{\prime }\right]\\ & \times p_{K}\left(\Delta r\right)d^{2}rd^{2}\Delta r \end{array}$$

where *η* and $\eta^{\prime }$ are dummy variables and the integrals with respect to *η* and $\eta^{\prime }$ represent convolutions with respect to *ɛ* and $\varepsilon^{\prime }$ , respectively. Noting that the convolution of two normal distributions yields a normal distribution with mean and variance equal to the sum of those of the initial distributions yields

(E4) $$\begin{array}{cc} I & =\int_{A}\int_{A}N_{B+C}\left(\varepsilon ;r,r-\Delta r\right)\\ & \times N_{B+C}\left(\varepsilon^{\prime };r+\tau ,r+\tau -\Delta r\right)p_{K}\left(\Delta r\right)d^{2}rd^{2}\Delta r. \end{array}$$

Performing the integration with respect to **r** yields

(E5) $$\begin{array}{cc} I & =\int_{A}N_{B+C}\left(\varepsilon ;\tau ,\tau -\tau^{\prime }\right){*}_{\tau }N_{B+C}\left(\varepsilon^{\prime };\tau ,\tau -\tau^{\prime }\right)\\ & \times p_{K}\left(\tau^{\prime }\right)d^{2}\tau^{\prime }. \end{array}$$

Integrating *ɛ* and $\varepsilon^{\prime }$ over energy bins *i* and *j*, respectively, yields

(E6) $$I=\int_{A}\Phi_{B+C,i}\left(\tau ,\tau -\tau^{\prime }\right){*}_{\tau }\Phi_{B+C,j}\left(\tau ,\tau -\tau^{\prime }\right)p_{K}\left(\tau^{\prime }\right)d^{2}\tau^{\prime }.$$

Integrating the preceding equation over the energy spectrum yields the third term in Eq. (36).
