# Supplementary material for: Frequency‐dependent signal and noise in spectroscopic x‐ray imaging
Source: Med Phys. 2020 Apr 22;47(7):2881–901. doi: 10.1002/mp.14160 (PMC7496729; doi:10.1002/mp.14160)
Supplement: Supplementary file 1 — Data S1: Supplemental material. [file MP-47-2881-s001.pdf]

Supplementary Material for "Frequency-dependent signal and noise in spectroscopic x-ray imaging"

Jesse Tanguay,[a,*] Jinwoo Kim,[b] Ho Kyung Kim,[b] Kris Iniewski,[c] and Ian A. Cunningham[d,e,f]
[a]*Department of Physics, Ryerson University, Toronto, Ontario, Canada, M5B 2K3;*
[b]*School of Mechanical Engineering, Pusan National University, Jangjeon-dong, Geumjeong-gu, Busan 609-735, Republic of Korea*
[c]*Redlen Technologies, Saanichton, British Columbia, Canada*
[d]*Imaging Research Laboratories, Robarts Research Institute, The University of Western Ontario, London, Ontario, Canada, N6A 5B7;*
[e]*Department of Medical Biophysics, Schulich School of Medicine & Dentistry, The University of Western Ontario, London, Ontario, Canada, N6A 5C1;*
[f]*Biomedical Engineering, Schulich School of Medicine & Dentistry, The University of Western Ontario, London, Ontario, Canada, N6A 5C1; and*
**Corresponding Author: 1-416-979-5000 ext. 2405, jesse.tanguay@ryerson.ca*

We describe the Matlab-based simulations of x-ray interactions in cadmium telluride (CdTe) detectors used in our manuscript. A flow-chart of the simulation is illustrated in Fig. 1.

For each simulated photon, a Bernoulli random variable (RV), $\tilde{\alpha}$, is sampled to determine whether or not the photon interacts within the CdTe detector. The probability of interaction is calculated from the quantum efficiency, α:

$$\alpha = 1 - e^{-\mu_{\text{tot}} L} \tag{1}$$

where μ_{tot} represents the linear attenuation coefficient of CdTe and L represents the converter thickness. When an interaction occurs, a second Bernoulli RV is sampled to determine whether or not the interaction occured with a Cd atom or aTe atom. The probability of interaction with Cd given an interaction is

$$\left(\begin{array}{c} \text{Probability of interaction with Cd} \\ \text{given an interaction} \end{array} \right) = \frac{\mu_{\text{Cd}}}{\mu_{\text{tot}}} \tag{2}$$

where μ_{Cd} represents the linear attenuation coefficient of Cd.

Interactions with Cd. When an interaction occurs with Cd, a third Bernoulli RV is sampled to determine whether or not the interaction was photoelectric or Compton. The probability of a PE interaction was calculated as

$$\left(\begin{array}{c} \text{Probability of PE interaction} \\ \text{given an interaction with Cd} \end{array} \right) = \frac{\mu_{\text{Cd,PE}}}{\mu_{\text{Cd}}}. \tag{3}$$

In the event of a Compton interaction, the energy of the recoil electron is assumed to be deposited locally; the energy carried away by the scattered photon is ignored. The number of secondary quanta liberated at the site of the Compton interaction, $\tilde{g}_{\text{Co}}$, is sampled from a Poisson distribution with mean value T/w where T represents the kinetic energy of the recoil electron and w is the energy required to liberate an e-h pair. Each secondary quantum is then relocated relative to the site of generation by sampling the charge-sharing kernel ($p_{\text{CS}}(\mathbf{r})$) described in the manuscript.

In the event of PE interaction, a fourth Bernoulli RV is sampled to determine whether or not a K-shell characteristic photon is produced. The probability of K-shell emission is calculated from the product of the K-shell participation fraction (P_K) and the fluorescence yield (ω_K). When a characteristic photon is not produced, the number of secondary quanta liberated at the site of x-ray interaction ($\tilde{g}_{\text{A}}$) is sampled from a Poisson distribution with mean value E_0/w. Each secondary quantum is then relocated relative to the site of generation by sampling $p_{\text{CS}}(\mathbf{r})$. When a characteristic photon is produced, the number of secondary quanta liberated locally ($\tilde{g}_{\text{B}}$) is sampled from a Poisson distribution with mean $(E_0 - E_K)/w$ where E_K is the average energy of K-shell transitions. The polar (θ) and azimuthal (ϕ) angles of the characteristic photon are sampled using the distributions described by Hajdok *et al.*[1] The radial distance ($\tilde{l}$) at which a the characteristic photon is reabsorbed is sampled from an exponential distribution:

$$p_l\left(l\right) = \mu_{\text{tot}} e^{-\mu_{\text{tot}} l} \tag{4}$$

where $p_l(l)$ represents the probability density function for l. The x, y, and z coordinates of the interaction are then calculated from l, θ, and ϕ. If $z \in [0, L]$, the number of secondary quanta liberated at the reabsorption site is sampled from a Poisson distribution with mean E_K/w.

For each detector element, the number of secondaries from each path is then summed. For each element, a zero-mean normal distribution with variance σ^2_{add} is then sampled to simulated electronic noise. The number of secondary quanta is then compared to a threshold to increment counts in pre-specified energy bins.

Interactions with Te. A similar approach is used to simulate interactions in Te, with the exception that we account for the production of Cd x-rays following interactions between Te x-rays and Cd atoms. As such, Compton interactions and PE interactions for which characteristic photon are not produced are treated using the exact same approach as that for Cd atoms, with the replacement of Cd properties with those of Te. For interactions that produce a characteristic photon, everything

up to and including the reabsorption of the characteristic photon is treated the same as for interactions with Cd. When a reabsorption occurs, a Bernoulli RV is sampled to determine whether or not the interaction is with Te or Cd. If the characteristic photon is reabsorbed with a Te atom, then the number of secondary quanta liberated at the reabsorption site is sampled from a Poisson distribution with mean E_K/w where E_K is average energy of K-shell transitions in Te. If the characteristic photon is reabsorbed in a Cd atom, then a Bernoulli RV is sampled to determine if a Cd characteristic photon is produced. If characteristic emission occurs, then the probability of reabsorption, location of reabsorption, and number secondaries liberated at primary and secondary interaction sites is sampled using the approach described above. Summing of quanta from all paths, electronic noise, and thresholding are then simulated using the same approach as that described for Cd.

REFERENCES

[1] G. Hajdok, J. Yao, J. J. Battista, and I. A. Cunningham, "Signal and noise transfer properties of photoelectric interactions in diagnostic x-ray imaging detectors," *Med. Phys.* **33**, pp. 3601–3620, Oct 2006.

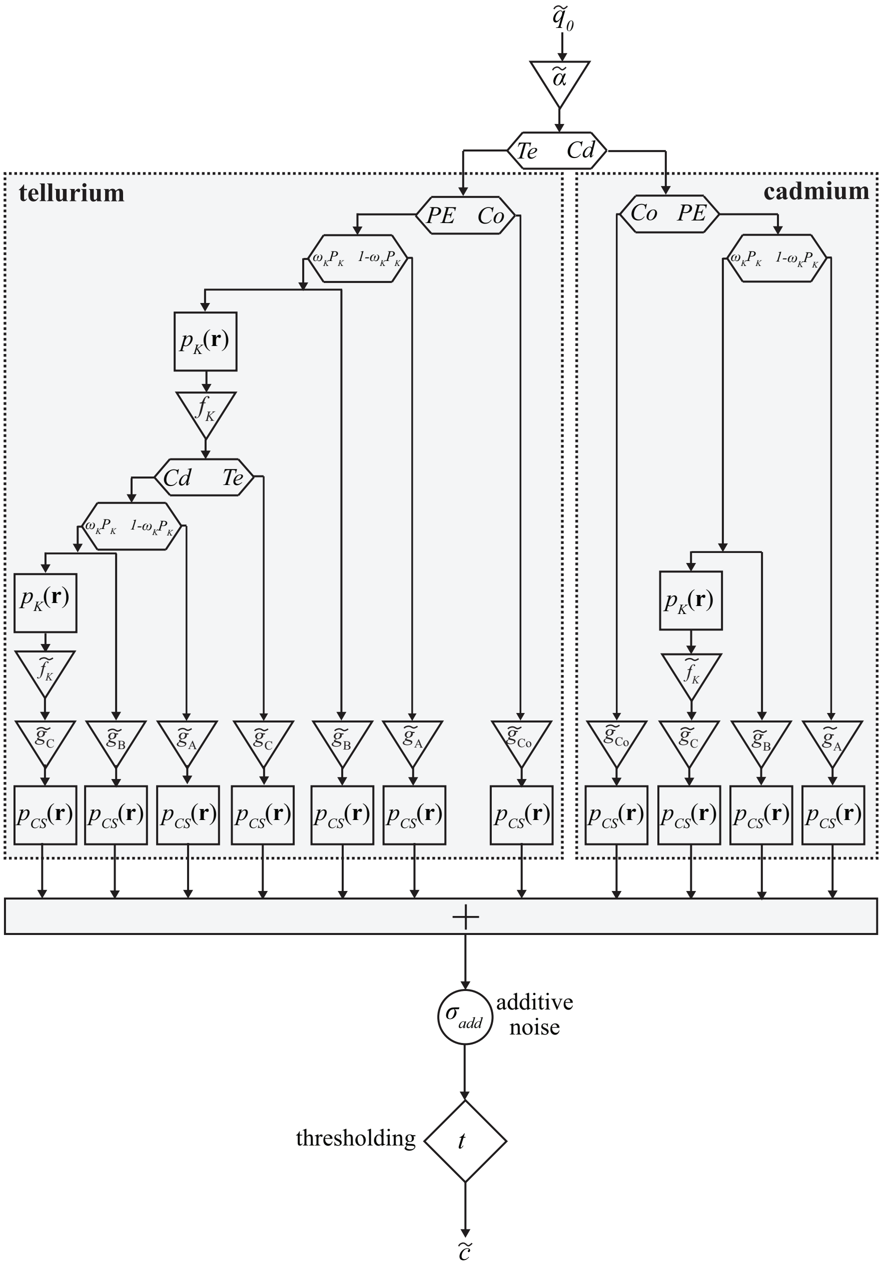

Figure 1. Schematic illustration of the framework used to simulate x-ray interactions in CdTe photon-counting detectors.
